# Identification of neural alterations in patients with Crohn’s disease with a novel multiparametric brain MRI-based radiomics model

**DOI:** 10.1186/s13244-024-01859-6

**Published:** 2024-11-29

**Authors:** Ruo-nan Zhang, Yang-di Wang, Hai-jie Wang, Yao-qi Ke, Xiao-di Shen, Li Huang, Jin-jiang Lin, Wei-tao He, Chen Zhao, Zhou-lei Li, Ren Mao, Ye-jun Wang, Guang Yang, Xue-hua Li

**Affiliations:** 1 Department of Radiology, The First Affiliated Hospital of Sun Yat-Sen University, Guangzhou, 510080 People’s Republic of China; 2 Shanghai Key Laboratory of Magnetic Resonance, East China Normal University, Dongchuan Road, Minhang District, Shanghai, 200241 People’s Republic of China; 3 MR Research Collaboration Team, Siemens Healthineers, Guangzhou, People’s Republic of China; 4 Department of Gastroenterology, The First Affiliated Hospital of Sun Yat-Sen University, Guangzhou, 510080 People’s Republic of China; 5 Youth Innovation Team of Medical Bioinformatics, Shenzhen University Medical School, Shenzhen, 518060 People’s Republic of China; 6 Department of Cell Biology and Genetics, College of Basic Medicine, Shenzhen University Medical School, Shenzhen, 518060 People’s Republic of China

**Keywords:** Crohn’s disease, Brain MRI, Gut-brain axis, Radiomics, Multiomics

## Abstract

**Objectives:**

Gut-brain axis dysfunction has emerged as a key contributor to the pathogenesis of Crohn’s disease (CD). The elucidation of neural alterations may provide novel insights into its management. We aimed to develop a multiparameter brain MRI-based radiomics model (RM) for characterizing neural alterations in CD patients and to interpret these alterations using multiomics traits.

**Methods:**

This prospective study enrolled 230 CD patients and 46 healthy controls (HCs). Participants voluntarily underwent brain MRI and psychological assessment (*n* = 155), blood metabolomics analysis (*n* = 260), and/or fecal 16S rRNA sequencing (*n* = 182). The RM was developed using 13 features selected from 13,870 first-order features extracted from multiparameter brain MRI in training cohort (CD, *n* = 75; HCs, *n* = 32) and validated in test cohort (CD, *n* = 34; HCs, *n* = 14). Multiomics data (including gut microbiomics, blood metabolomics, and brain radiomics) were compared between CD patients and HCs.

**Results:**

In the training cohort, area under the receiver operating characteristic curve (AUC) of RM for distinguishing CD patients from HCs was 0.991 (95% confidence interval (CI), 0.975–1.000). In test cohort, RM showed an AUC of 0.956 (95% CI, 0.881–1.000). CD-enriched blood metabolites such as triacylglycerol (TAG) exhibited significant correlations with both brain features detected by RM and CD-enriched microbiota (e.g., *Veillonella*). One notable correlation was found between *Veillonella* and Ctx-Lh-Middle-Temporal-CBF-p90 (*r* = 0.41). Mediation analysis further revealed that dysbiosis, such as of *Veillonella*, may regulate the blood flow in the middle temporal cortex through TAG.

**Conclusion:**

We developed a multiparameter MRI-based RM that characterized the neural alterations of CD patients, and multiomics data offer potential evidence to support the validity of our model. Our study may offer clues to help provide potential therapeutic targets.

**Critical relevance statement:**

Our brain-gut axis study developed a novel model using multiparameter MRI and radiomics to characterize brain changes in patients with Crohn’s disease. We validated this model’s effectiveness using multiomics data, making it a potential biomarker for better patient management.

**Key Points:**

- Utilizing multiparametric MRI and radiomics techniques could unveil Crohn’s disease’s neurophenotype.
- The neurophenotype radiomics model is interpreted using multiomics data.
- This model may serve as a novel biomarker for Crohn’s disease management.

**Graphical Abstract:**

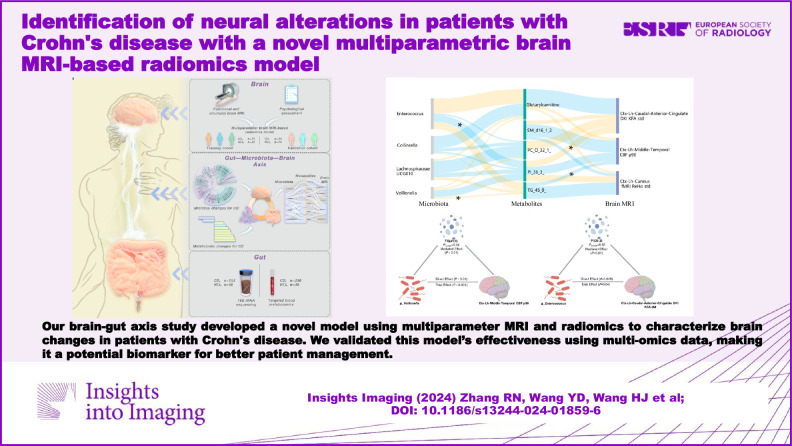

## Introduction

Crohn’s disease (CD) is a chronic inflammatory bowel disease (IBD), characterized by clinical symptoms such as abdominal pain, persistent diarrhea, unintended weight loss, chronic fatigue, and/or diminished appetite [1]. IBD, such as CD, predisposes individuals to mental disorders, particularly depression and anxiety [2]. The prevalence of comorbid depression and anxiety in individuals with IBD ranges from 20 to 40% [3]. The presence of anxiety and depression in turn exacerbates intestinal symptoms [4]. The gut-brain axis serves as a crucial physiological connection between the central nervous system and gastrointestinal tract, and accumulating evidence indicates that it is involved in the pathogenesis, making it a promising target for managing CD [5]. However, the neural alterations in CD patients remain unclear, impeding the development of corresponding treatment strategies [6].

Brain magnetic resonance imaging (MRI) is useful in describing neural alterations and elucidating the relationship between the gut and the brain in CD patients [7]. Previous studies have connected brain features such as functional and structural alterations in the amygdala and insula with mood, cognition, and intestinal conditions in CD [8–10]. Aberrant functional connectivity (FC) of the amygdala may be involved in the processing of visceral pain sensation and emotion in CD [9]; while insula-centered structural and/or functional changes may be associated with abnormal visceral sensory processing and related emotional responses [10]. However, most of these studies only used a limited number of MRI parameters, such as those derived solely from diffusion tensor imaging [8], resting-state blood-oxygen-level-dependent (BOLD) [11, 12], BOLD with T1-weighted structural sequence [10, 13, 14], or with proton magnetic resonance spectroscopy parameters [15]. This limitation may have constrained the accuracy of pathophysiological interpretation. In contrast, more comprehensive multiparameter neuroimaging may address these limitations and provide novel insights into complex neural processes.

Radiomics, which extracts numerous features from medical images and builds machine-learning models for diagnosis, has been widely used in the medical field [16]. We hypothesized that radiomics, combined with multiparameter brain MRI, holds promise for comprehensive characterization of brain features, and can overcome the limitations of insufficient data mining and interpretation.

Furthermore, recent evidence has revealed significant associations among the gut microbiome, metabolite disorders, and neurological disorders in other diseases, such as amnestic mild cognitive impairment and irritable bowel syndrome [17, 18], suggesting the feasibility of employing a multiomics approach to investigate the underlying association of the gut-brain axis in CD.

This prospective study aimed to develop and validate a radiomics model (RM) based on multiparameter brain MRI data for the characterization of the neurophenotype of CD patients and to interpret this RM using multiomics data, including brain radiomics, fecal 16S rRNA sequencing, and blood metabolomics.

## Materials and methods

### Subjects

This prospective, observational, single-center study was approved by the Institutional Ethics Review Board of The First Affiliated Hospital of Sun Yat-sen University (No. [2021]215-2). Informed consent was obtained from all participants.

**Fig. 1:**
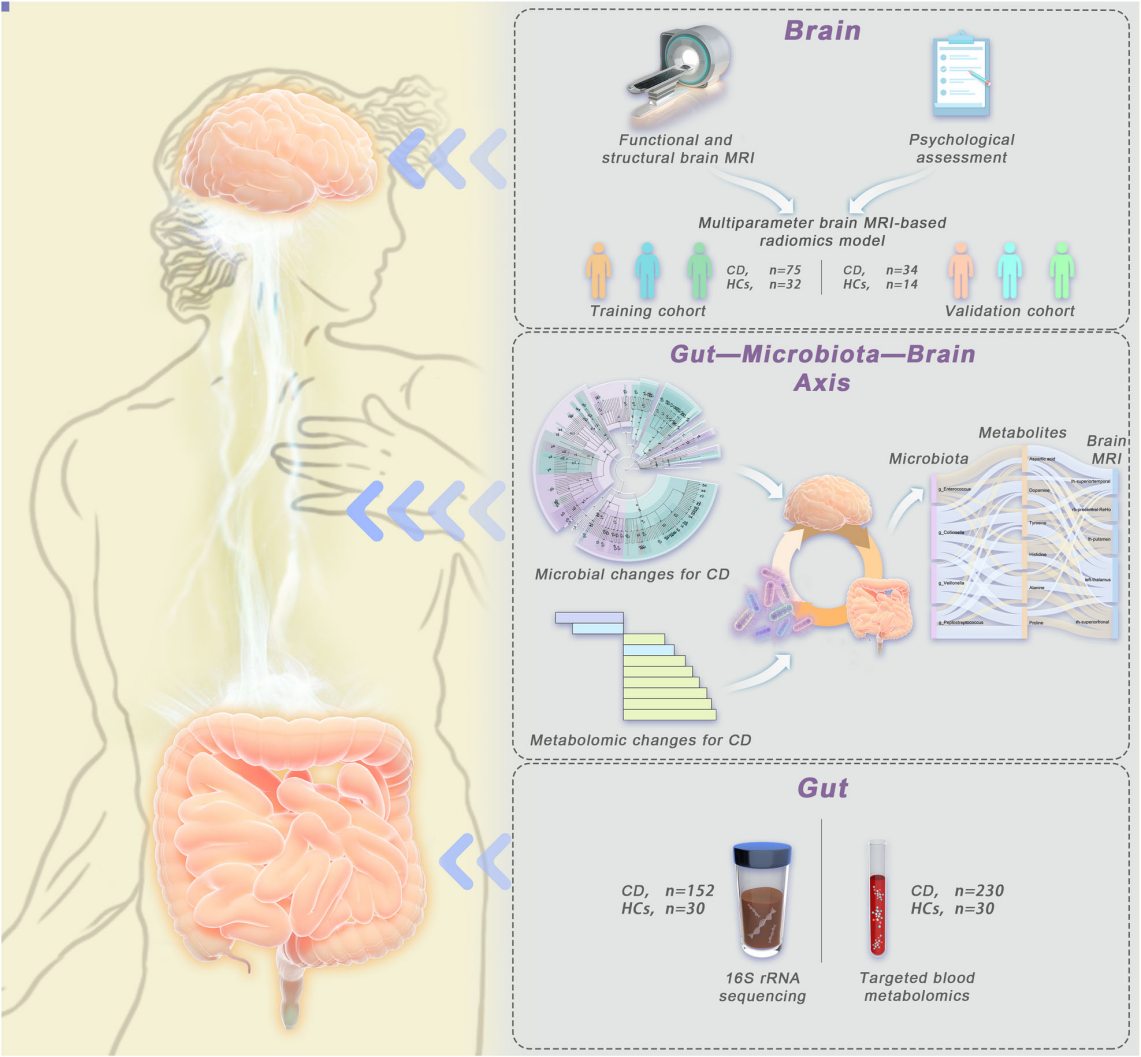
Overview of study design and analyses. We recruited a total of 276 participants (CD, *n* = 230; HCs, *n* = 46) and collected fecal samples, blood samples, multiparameter brain MRI data, and responses to a psychological questionnaire. Subsequently, a multiomics analysis of the Gut-brain axis was conducted to investigate the brain features of CD and the underlying mechanisms. (CD, Crohn’s disease; HCs, healthy controls; 16Sr RNA, 16S ribosomal ribonucleic acid)

From June 2021 to May 2023, 243 CD patients and 46 healthy controls (HCs) were recruited. To investigate the gut-brain axis multiomics correlation, we enrolled CD patients who voluntarily provided blood or stool samples, underwent brain MRI scans, or participated in any combination of these three procedures. The inclusion criteria for CD patients and HCs were as follows: (a) available fecal or blood samples or complete brain MRI data (protocol see below), (b) aged 18–45 years (for minimizing the potential impact of older age on brain structure or function, as well as younger age on limited cooperation during MRI scans and psychological scale surveys [19–21]), and (c) right-handedness (for minimizing the potential impact of handedness on brain function [22]). The exclusion criteria were as follows: (a) antibiotic, probiotic, or prebiotic use in the three months before inclusion (for minimizing the potential impact on gut microbiota [23, 24]); (b) other concomitant organic/functional digestive diseases; or (c) in those who had received brain MRI, one of the following: (1) a history of neurosurgery, head injury, cerebrovascular disease, or brain trauma with loss of consciousness; (2) central nervous system drug or antidepressant use within 3 months before inclusion; (3) temporary depression caused by adverse life events; (4) claustrophobia; or (5) metal implants. The study protocol is presented in Fig. 1, and the participants’ recruitment process is shown in Supplementary Fig 2.

## Development and validation of multiparameter brain MRI-based RM

### Brain MRI acquisition

A total of 155 participants who underwent brain MRI were randomly assigned to two cohorts at a ratio of 7:3: training cohort (CD, *n* = 75; HCs, *n* = 32) and test cohort (CD, *n* = 34; HCs, *n* = 14). MRI was acquired in a 3-T MRI scanner (MAGNETOM Prisma; Siemens Healthineers, Erlangen, Germany) with a 64-channel phased-array head coil. The MRI sequences included resting-state BOLD imaging (for reflecting the level of spontaneous neural activity at each voxel) [25], diffusion spectrum imaging (DSI, for assessing microstructure properties of tissue) [26], quantitative susceptibility mapping (for evaluating iron variations) [27], arterial spin labeling (for quantifying cerebral perfusion) [28], and unenhanced T1-weighted imaging (T1WI, for providing anatomical details) [29]. The combination of these sequences was employed to achieve a more comprehensive brain characterization of patients with CD. The time required to complete the whole brain examination was 50 min. The protocol parameters are described in Supplementary Table 1.

### Data preprocessing, brain region segmentation, and radiomics feature extraction

**Fig. 2:**
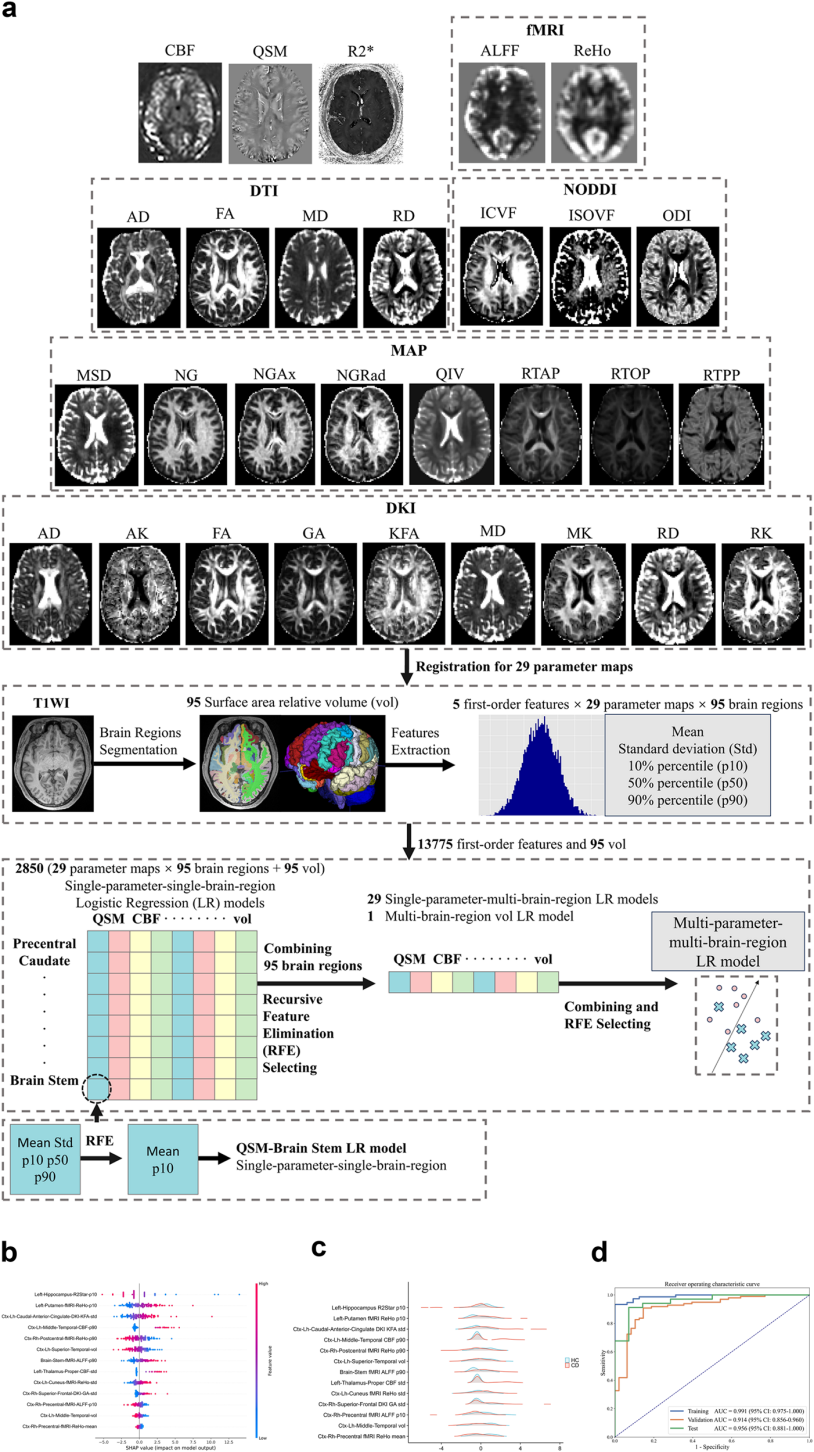
Development and validation of the multiparameter brain MRI-based RM. **a** Flowchart illustrating the process of multiparameter brain MRI acquisition, image preprocessing, brain region segmentation, radiomics feature extraction and selection, and radiomics model development. **b** The SHAP summary plot of 13 brain features in the RM (visual interpretation of the RM). The general importance of the 13 features is demonstrated in descending order of their importance, from highest to lowest (see Table 3). The horizontal location indicates whether the effect of that value is associated with a higher or lower prediction, and the color represents the level of that variable (high = red; low = blue) for that observation. For example, the increase in Left-Hippocampus-R2Star-p10 had a negative impact and drove the prediction of the brain features of HCs, whereas the increase in Left-Putamen-fMRI-ReHo-p10 had a positive impact and drove the prediction of the brain features of CD patients. The naming scheme used for the 13 brain radiomics features includes three main components: the brain region, the MRI sequence or parameter, and the first-order radiomics feature. In the depiction of the brain region, the abbreviation “ctx” represents the cortex in the specific brain region, while “l” and “r” represent the left and right sides, respectively; “h” represents the head. **c** The ridge plots display the data distributions of these 13 radiomics features in CD patients and HCs. Other detailed statistical information is shown in Table 3. **d** ROC curve of the RM for distinguishing CD patients from HCs. The high AUCs of the RM in the training, validation, and test cohorts indicate that the RM effectively characterized the brain features of CD patients. The results of the validation cohort were generated during the modeling process, wherein a fivefold cross-validation was conducted using the training cohort to determine the final set of radiomic features. (CBF, cerebral blood flow; QSM, quantitative susceptibility mapping; fMRI, functional MRI; ALFF, amplitude of low-frequency fluctuations; ReHo, regional homogeneity; DTI, diffusion tensor imaging; AD, axial diffusivity; FA, fractional anisotropy; MD, mean diffusivity; RD, radial diffusivity; NODDI, neurite orientation dispersion and density imaging; ICVF, intracellular volume fraction; ISOVF, isotropic volume fraction; ODI, orientation dispersion index; MAP, mean apparent propagator; MSD, mean squared displacement; NG, non-Gaussian; NGAx, axial non-Gaussian; NGRad, radial non-Gaussian; QIV, q-space inverse variance; RTAP, return-to-axis probability; RTOP, return-to-origin probability; RTPP, return-to-plane probability; DKI, diffusion kurtosis imaging; AK, axial kurtosis; GA, geodesic anisotropy; KFA, kurtosis fractional anisotropy; MK, mean kurtosis; RK, radial kurtosis; T1WI, T1-weighted imaging; std, standard deviation; p10, 10th percentile; p90, 90th percentile; SHAP, Shapley Additive Explanations; RM, radiomics model; AUC, area under the ROC curve; CI, confidence interval)

After MRI, the data were processed to generate 29 quantitative-parameter maps (Supplementary Material 1; Fig. 2a). First, to enhance the preservation of anatomical details, we automatically segmented brain T1WI with the FreeSurfer toolbox (version 6.0) [30] and using the FastSurfer approach [31] and the Desikan-Killiany atlas [32]. All parameter maps were then registered to T1WI using Elastix (https://elastix.lumc.nl/). Subsequently, the PyRadiomics (version 3.0.1, https://pyradiomics.readthedocs.io/) package was used to extract five first-order features, including the mean, standard deviation (std), 10th percentile (p10), 50th percentile (p50), and 90th percentile (p90), from each brain region on each map. Therefore, 13,775 first-order features (5 first-order features × 29 parameter maps × 95 brain regions) and 95 relative volume (vol) features of brain regions were extracted, resulting in 13,870 features for each subject. The image processing and feature extraction procedures were implemented within our in-house postprocessing tool, BrainQuanAll, which is based on Python code (version 3.8).

### Development and validation of the RM

Before modeling, the training cohort was balanced using upsampling to ensure an equal proportion of positive and negative samples in the dataset. The first-order radiomics features were standardized using the Z transformation. To reduce the dimensionality and select the best subset of features from the 13,870 features, a three-step strategy was used to develop a multiparameter-multibrain-region logistic regression (LR) model with an open-source software FeatureExplorer [33] (Fig. 2a; Supplementary Fig. 1; Supplementary Material 2). To ensure the robustness and generalization of the multiparameter model, fivefold cross-validation was performed with the training set to determine the final feature set. The radiomics score (rad-score) for each participant was calculated according to the final multiparameter-multibrain region model.

$${{\rm{The}}}\; {{\rm{final}}}\; {{\rm{RM}}}\; {{\rm{was}}}\!:{sigmoid}\left(\left(\sum _{k=0}^{n}{FeatureK}* {Coef}\right)+{bias}\right)$$

where “*FeatureK*” is value of the k-th brain feature retained in RM, ‘*Coef*” are values generated by RM for each brain feature, “*bias*” is the bias value of linear regression. Details of the model formula can be found in Supplementary Material 2.

In addition, to further test the performance of RM, we constructed a comprehensive brain-psychological-clinical LR model (Supplementary Material 3) to compare it with the single RM (using DeLong’s test). We utilized receiver operating characteristic (ROC) curves and the area under the ROC curve (AUC) to assess the performance of the prediction models. The classification threshold was obtained by maximizing Youden’s index in the training set, which was used to calculate accuracy (ACC), sensitivity (SEN), and specificity (SPE). Shapley Additive Explanations (SHAP) were used to visualize the models by evaluating the importance of each feature in the models [34]. The distribution of these features was displayed by ridge plots [35]. The statistical analysis was implemented using the open-source Python libraries SciPy and scikit-learn.

### Psychological questionnaire and clinical data collection

The 155 participants who underwent brain MRI completed the State-Trait Anxiety Inventory (STAI) [36], Perceived Stress Scale (PSS) [37], and Beck Depression Inventory (BDI) [38], which were used to assess their psychological conditions (Supplementary Material 4; Supplementary Tables 2–5).

Clinical data, including sex, age, education level, body mass index (BMI), smoking and alcohol history, were collected through a questionnaire. Both drinking and smoking history were treated as binary variables. Self-reported behaviors of past or present smoking and drinking were defined as smoking and drinking, respectively [39].

### The performance comparison between RM and comprehensive brain-psychological-clinical model

To further investigate the RM’s performance, we combined the psychological factors (including STAI, BDI, and PSS scores) and clinical factors (including alcohol history, BMI, and education level that were different significantly between CD and HCs) with RM to develop a comprehensive model and compared its performance with RM.

### Fecal 16S rRNA sequencing and blood metabolomics profiling

Fecal samples were subjected to 16S rRNA gene amplicon sequencing, with a focus on identifying the genera that were enriched in patients with CD compared with HC. Blood samples were used for targeted metabolomics profiling to detect 528 specific metabolites (Supplementary Material 5). The analyses of the gut microbiota and blood metabolites were performed in R statistical software (version 4.2.2). Microbial alpha diversity [40] was evaluated based on Chao1 [41], Simpson [42], Shannon [43], and Pielou indices [44]. Bray‒Curtis distances [45] and beta diversity [46] were also calculated. Definition and index introduction of Alpha and Beta diversity are shown in Supplementary Table 10.

### Statistical analysis

Statistical analyses were carried out using R statistical software. A two-sided *p*-value < 0.050 indicated statistical significance. Univariate analysis for variables was conducted using Student’s *t*-test, Mann–Whitney *U* test, or Chi-square test based on data distribution.

Wilcoxon rank-sum tests were performed to compare alpha diversity between CD patients and HCs. Permutational multivariate analysis of variance (PERMANOVA) was performed with the adonis function in Vegan (version 2.6) to assess the microbial variance explained by different groups (https://view.qiime2.org/). Linear discriminant analysis effect size (LEfSe) was performed to identify the differentially abundant taxa. After normalization of metabolomic data, principal component analysis (PCA) was conducted to observe their distribution within and between groups. Differentially abundant metabolites between groups were identified with univariate analysis and orthogonal partial least squares discriminant analysis (OPLS-DA).

Pairwise linear regression analysis was performed to investigate correlations within multiomics data in CD patients while adjusting for age, sex, and BMI. Only variables with *p* < 0.05 from the pairwise regression analysis are used for subsequent Pearson correlation and mediation analyses, to further explore associations within the multiomics data.

## Results

### The differences in clinical and psychological data between CD patients and HCs

Finally, 276 subjects were included (CD, *n* = 230; HCs, *n* = 46; Supplementary Fig. 2). Among these participants, 260 (CD, *n* = 230; HCs, *n* = 30) provided blood samples, 182 (CD, *n* = 152; HCs, *n* = 30) provided fecal samples, and 155 (CD, *n* = 109; HCs, *n* = 46) additionally underwent brain MRI and completed psychological questionnaires. All these data were collected within one week.

The demographic characteristics, clinical variables, and psychological questionnaire results of the participants are shown in Table 1. In addition to intestinal conditions, we also considered lifestyle factors such as smoking and alcohol consumption which could potentially influence brain structure or function [47]. Significant differences were observed between CD patients and HCs in educational level and psychological scores in both the training and test cohorts (all *p* < 0.05). The differences in alcohol consumption and BMI between CD patients and HCs were significant in the training cohort (both *p* < 0.01) but not in the test cohort (both *p* > 0.05).

**Table 1:** The characteristics of the study population

Characteristics	Total CD patients	Participants recruited for brain MRI scan					
		Training cohort (*n* = 107)		*p*-value	Test cohort (*n* = 48)		*p*-value
Number of participants	230	CD (*n* = 75)	HCs (*n* = 32)		CD (*n* = 34)	HCs (*n* = 14)	
Clinical variates							
Sex, *n* (male/female)	184/46	64/11	28/4	0.77	30/4	11/3	0.39
Age, years (mean ± SD)	30.30 ± 8.00	28.47 ± 7.38	30.56 ± 6.24	0.10	31.26 ± 6.25	26.43 ± 4.45	0.01
Education^#^ (median (IQR))	…	6 (5–6)	4 (4–5)	< 0.001	4 (3.75–5)	6 (5–6)	< 0.001
Smoking history, *n* (%)	21 (9.13%)	8 (10.67%)	7 (21.88%)	0.13	6 (17.65%)	0 (0)	0.10
Alcohol history, *n* (%)	27 (11.74%)	15 (20%)	17 (53.13%)	0.00	6 (17.65%)	5 (35.71%)	0.18
BMI (kg/m^2^) (mean ± SD)	19.96 ± 3.32	20.06 ± 3.25	22.86 ± 2.01	< 0.001	20.31 ± 3.60	22.26 ± 2.73	0.06
Psychological scales							
S-score (mean ± SD)	…	41.04 ± 10.11	29.41 ± 5.89	< 0.001	43.09 ± 10.73	29.21 ± 6.17	< 0.001
T-score (mean ± SD)	…	42.35 ± 9.73	31.94 ± 6.00	< 0.001	44.53 ± 10.52	30.07 ± 5.84	< 0.001
BDI (median (IQR))	…	9 (4–15)	4 (1–7)	< 0.001	8.5 (5–17.5)	1 (0–3)	< 0.001
PSS (mean ± SD)	…	37.14 ± 8.13	29.47 ± 5.96	< 0.001	37.32 ± 7.65	28.71 ± 6.82	< 0.001
Disease traits							
Disease duration, months (median (IQR))	48 (13.50–96)	40 (12–84)	…	…	90 (24–120)	…	< 0.01
Score of abdominal pain* (median (IQR))	…	3 (2–5)	…	…	2 (1–4.25)	…	0.11
CDAI, *n* (%)							0.52
Remission (< 150)	92 (40%)	25 (33.33%)	…	…	15 (44.12%)	…	…
Mild disease (150–220)	56 (24.30%)	21 (28%)	…	…	7 (20.59%)	…	…
Moderate disease (220–450)	81 (35.20%)	29 (38.67%)	…	…	12 (35.29%)	…	…
Severe disease (> 450)	1 (0.50%)	0 (0)	…	…	0 (0)	…	…
Disease location, *n* (%)							0.10
L1(terminal ileum ± cecum)	26 (11.30%)	11 (14.67%)	…	…	8 (23.53%)	…	…
L2 (colonic)	9 (3.91%)	1 (1.33%)	…	…	3 (8.82%)	…	…
L3 (ileocolic)	186 (80.87%)	60 (80%)	…	…	23 (67.65%)	…	…
L4 (upper gastrointestinal tract)	9 (3.91%)	3 (4%)	…	…	0 (0)	…	…
Disease behavior, *n* (%)							0.61
Non-stricturing, non-penetrating	89 (38.70%)	25 (33.33%)	…	…	14 (41.18%)	…	…
Intestinal stricture	70 (30.43%)	26 (34.57%)	…	…	12 (35.29%)	…	…
Intestinal penetration	71 (30.87%)	24 (32%)	…	…	8 (23.53%)	…	…
Perianal diseases, *n* (%)							0.30
None	87 (37.83%)	23 (30.67%)	…	…	14 (41.18%)	…	…
Anal fistula	115 (50%)	45 (60%)	…	…	15 (44.12%)	…	…
Perianal abscess	28 (12.17%)	7 (9.33%)	…	…	5 (14.71%)	…	…

Data are presented as mean ± SD for data that follow a normal distribution, or as median (IQR) for data that do not follow a normal distribution

*CD* Crohn’s disease, *HC* healthy control, *BMI* body mass index, *S-score* state score of State-Trait Anxiety Inventory, *T-score* trait score of State-Trait Anxiety Inventory, *PSS* perceived stress scale, *BDI* Beck depression inventory, *SD* standard deviation, *IQR* interquartile range, *CDAI* Crohn’s disease activity index

^#^ The educational level is assessed on a scale of 1 to 6: primary school (score 1), junior high school (score 2), senior high school (score 3), junior college (score 4), undergraduate (score 5) and postgraduate (score 6)

* Visual Analogue Scale is used to assess the severity of abdominal pain in CD patients, ranging from score 0 to 10. A score of 0 indicates the absence of pain, while a score of 10 represents excruciating pain

### The brain features of CD patients identified by RM differ from those of HCs

Thirteen brain radiomics features were finally used to develop RM. Compared with previously published literature (see Table 2 for details), among the 13 brain features, five features (Left-Hippocampus-R2Star-p10, Ctx-Lh-Caudal-Anterior-Cingulate-DKI-KFA-std, Ctx-Lh-Middle-Temporal-CBF-p90, Left-Thalamus-Proper-CBF-std, and Ctx-Rh-Superior-Frontal-DKI-GA-std) in our study were identified as novel specific markers for CD patients. These features have not yet been reported in the existing literature for CD patients. Their general importance in RM is shown in Table 3 and SHAP plot (Fig. 2b). Specifically, Left-Hippocampus-R2Star-p10, Left-Putamen-fMRI-ReHo-p10, and Ctx-Lh-Caudal-Anterior-Cingulate-DKI-KFA-std were the three most important features. Ridge plot shows the differences in data distributions for the same brain feature between HCs and CD patients (Fig. 2c). For example, in the Left-Hippocampus-R2Star-p10 feature, CD patients exhibited a lower mean value compared to that of HCs, suggesting a potential association between a decreasing value of this feature and CD.

**Table 2:** The pathophysiologic interpretations of the 13 brain features in radiomics model

Brain features	Physiological function of the brain region	Reported changes of the brain region in CD	Clinical significance of the MRI parameter	Reported changes of the MRI parameter in CD	Speculative interpretation of brain features in CD
**Left-Hippocampus- R2Star-p10**	Hippocampus: The brain limbic system, responsible for adult neurogenesis, emotional control, and cognition. (*PMID: 31969694*)	Alterations in gray matter volume and functional activity of the hippocampus in CD patients compared to healthy volunteers. (*PMID: 29204113*) Experimental colitis in animals with IBD affects hippocampal neurogenesis and innate immune cell responses. (*PMID: 31969694*)	R2 Star: In vivo detection of iron deposition in brain tissue. (*PMID: 26445114*)	--*	The R2 Star in hippocampus may serve as a biomarker for detecting cognitive dysfunction or neurogenesis as well as innate immune cell responses in CD, by reflecting iron deposition.
Left-Putamen-fMRI-ReHo-p10	Putamen: Part of the striatum, the major input source of the basal ganglia; associated with integrating multiple sensory modalities and coordinating behavioral responses. (*PMID: 35185768*)	Inactive CD patients exhibited lower ReHo values in the putamen compared with healthy controls. (*PMID: 35185768*)	ReHo: The synchronization of fluctuations among adjacent voxels of blood oxygenation level-dependent signals to provide information about local activity. (*PMID: 35185768*)	As described in the section of “Reported changes of the brain region in CD” of this brain feature.	Lower ReHo values in CD patients may be related to psychological distress in these patients as this region is known to play a role in affective disorders.
**Ctx-Lh-Caudal-Anterior-Cingulate- DKI-KFA-std**	Caudal anterior cingulate gyrus: Part of a distributed attentional network; a crucial region in sensory and cognitive research, involving pain modulation and related emotional processing, such as anxiety, depression, and fear. (*PMID: 37286175*)	Peripheral inflammation of animal colitis model induced microglial and glutamatergic neuronal activation in the anterior cingulate cortex. Inhibition of glutamatergic neurons or depletion of microglia in the anterior cingulate cortex mitigates visceral pain, and the latter can also prevent depressive-like behaviors. (*PMID: 37286175*)	DKI KFA: Provides a concise representation of the directional variation in the degree of non-Gaussian diffusion; reflects tissue-diffusion complexity indicated injured white matter fiber integrity. (*PMID: 27041679*)	--*	The increased KFA in caudal anterior cingulate indicates a stronger connection among brain areas. Pain and related emotional processing, such as anxiety, depression and fear can cause sustained tension on this brain lobe.
**Ctx-Lh-Middle-Temporal-CBF-p90**	Temporal middle gyrus: Speech and hearing processing. (*PMID: 35185768*)	Active CD patients exhibited higher ReHo values in the temporal middle compared with HCs. (*PMID: 35185768*)	CBF: Provides brain oxygen information, which is essential to brain metabolism. Abnormal CBF may indicate cerebral microvascular dysfunction. (*PMID: 30358242*)	--*	The increased CBF in middle temporal may represent increased activity of these regions in an effort to bolster cognitive performance, and may suggest the adjustment to reduced nociceptive input or reflect an inherent susceptibility to heightened disease activity.
Ctx-Rh-Postcentral- fMRI-ReHo-p90	Cortex of Postcentral gyrus: A sensory area that explains various sensory stimuli; a primary receptor for general bodily feeling of touch, such as temperature and pain. (*PMID: 35185768*)	Compared with the HCs, CD patients exhibited lower ReHo values in the postcentral gyrus. The involvement of this brain region may contribute to the development of CD. (*PMID: 35185768*)	ReHo: As described in the section of “Clinical significance of the MRI parameter” of brain feature namely “Left-Putamen-fMRI-ReHo- p10”.	As described in the section of “Reported changes of the brain region in CD” of this brain feature.	Decreased ReHo values in the postcentral gyri cortex in CD patients may associated with the abnormal bodily feeling such as abdominal pain.
Ctx-Lh-Superior- Temporal-vol	Superior temporal gyrus: Associated with auditory processing, including language. As an important structure in the pathway, which are involved in social cognition processes and the perception of emotions in facial stimuli. (*PMID: 26843641* *PMID: 12724168* *PMID: 19699306*)	Joint independent component analysis detected structural alterations of temporal regions with voxel-based morphometry. It may act as one part of controls and patients point toward key hubs of the so-called default mode network (DMN), which is thought to mediate several discrete functions, including processing of self-referential information and affect, internal mentation and memory. (*PMID: 33368950*)	Vol: Cortical volume has been identified as a significant marker of brain pathology. (*PMID: 29212177*)	Negative correlations were found between the gray matter volumes in several brain regions (e.g., the insula and caudal anterior cinaulate) and the severity of abdominal pain in CD patients. (*PMID: 29212177*)	The alterations of volume in superior-temporal gyrus may contribute to the social anxiety disorder of CD patients. In general, the mechanism underlying cortical volume changes may be attributed to frequent and chronic nociceptive input and the subsequent functional reorganization and plasticity of the brain. The lower volumes could be related to a decrease in the size of neuronal somata, cell atrophy, or a decrease in intra-cortical axonal architecture (i.e., synaptic loss)
Brain-Stem-fMRI- ALFF-p90	Brain stem: The vital center for physiological activities in the human body, encompassing a multitude of functions, including the regulation of digestive systems.	--*	fMRI ALFF: The amplitude of low‐frequency fluctuations (ALFF) reflects the level of spontaneous activity at each voxel. ALFF is a classic metric that has mostly been calculated to determine abnormal brain activity precisely, with the energy of a time series being broken down into sets of stationary sinusoidal functions of different frequencies. (*PMID: 31451898*)	Patients with the active CD exhibited higher ALFF in several brain regions (e.g., the caudal anterior cingulate hippocampus, insula, superior-frontal cortex, precuneus, and parahippocampus). (*PMID: 35600612* *PMID: 29464530*)	The development and progression of intestinal inflammation or abdominal pain in CD patients may contribute to the increased level of spontaneous activity in the brain stem, as indicated by an increase in ALFF.
**Left-Thalamus-Proper- CBF-std**	Thalamus: a nuclear complex and relay center between the cerebral cortex and several subcortical brain regions located in the diencephalon, supporting both sensory and motor mechanisms. (*PMID: 12192499*)	CD patients had significantly stronger activations than controls in the thalamus. The volume of thalamus in IBD patients was smaller than that in control group. (*PMID: 27132547* *PMID: 34734248*)	CBF: As described in the section of “Clinical significance of the MRI parameter” of brain feature namely “Ctx-Lh-Middle-Temporal-CBF- p90”.	--*	The increased CBF in thalamus may indicate heightened activity in this region, which is recognized for its involvement in processing and regulating sensory, cognitive, emotional aspects of pain, as well as threat assessment.
Ctx-Lh-Cuneus-fMRI- ReHo-std	Cuneus: Correlates with sympathetic nervous system activity and processing of the autonomic subdimension. Plays a role in the perceptions of bodily expressions, threatening or fear-inducing signals. Interconnected with the default mode network (regions involved in resting-state brain activity) and limbic regions for vigilance, attention, motivation and arousal. (*PMID: 20723605* *PMID: 21255659*)	The reduction of nodal betweenness centrality was detected in the cuneus in CD patients through the building of topological properties of networks, suggesting that CD is accompanied by alterations in both global network organization and regional connectivity. (*PMID: 35111602*)	fMRI ReHo: As described in the section of “Clinical significance of the MRI parameter” of brain feature namely “Left-Putamen-fMRI-ReHo- p10”.	As described in the section of “Reported changes of the brain region in CD” of brain feature namely “Ctx-Rh-Postcentral-fMRI-ReHo- p90”.	Higher ReHo values in the cuneus of CD patients may be related to affective disorders, such as vigilance, arousal, attention, perceptions of fear, sensory and visuospatial transformation.
**Ctx-Rh-Superior-Frontal-DKI-GA-std**	Frontal regions: Involved in early auditory encoding. (*PMID: 33368950*)	Decreased gray matter volume in the frontal in CD patients. The structural alterations in superior-frontal gyrus in CD patients detected by ioint independent component analysis. (*PMID: 22998431* *PMID: 33368950*)	DKI GA (Geodesic Anisotropy): Measures the distance of a diffusion tensor to the nearest isotropic tensor, computed intrinsically on the manifold of positive-definite symmetric diffusion tensors. (*PMID: 30546820*)	--*	The increased GA in superior frontal of CD patients may indicate the alteration of brain fiber microstructure and abnormal connection of fronto-temporal auditory networks.
Ctx-Rh-Precentral- fMRI-ALFF-p10	Precentral gyrus: The site of the premotor cortex, which is involved in the sensorimotor network and is responsible for the execution and control of voluntary movements through the corticospinal tract. Inextricably involved in emotion control. (*PMID: 25001084*)	Inactive CD patients exhibited lower ReHo values in the precentral. (*PMID: 35185768*)	fMRI ALFF: As described in the section of “Clinical significance of the MRI parameter” of brain feature namely “Brain-Stem-fMRI-ALFF-p90”.	As described in the section of “Reported changes of the MRI parameter in CD” of brain feature namely “Brain-Stem-fMRI- ALFF-p90”.	The decreased ALFF in precentral gyrus of CD patients may be associated with sensorimotor and emotional dysfunctions.
Ctx-Lh-Middle-Temporal-vol	As described in the section of “Physiological function of the brain region” of brain feature namely “Ctx-Lh-Superior- Temporal-vol”.	As described in the section of “Reported changes of the brain region in CD” of brain feature namely “Ctx-Lh-Superior-Temporal- vol”.	As described in the section of “Clinical significance of the MRI parameter” of brain feature namely “Ctx-Lh-Superior-Temporal-vol”.	As described in the section of “Reported changes of the MRI parameter in CD” of brain feature namely “Ctx-Lh-Superior- Temporal-vol”.	The volume in middle temporal was decreased in CD patients compared with healthy people, which may be related to a decrease in the size of neuronal somata, cell atrophy, or a decrease in intra-cortical axonal architecture (i.e., synaptic loss), and may have acted as the adaptations to decreased nociceptive input.
Ctx-Rh-Precentral- fMRI-ReHo-mean	As described in the section of “Physiological function of the brain region” of brain feature namely “Ctx-Rh-Precentral- fMRI-ALFF-p10”.	As described in the section of “Reported changes of the brain region in CD” of brain feature namely “Ctx-Rh-Precentral-fMRI-ALFF- p10”.	ReHo: As described in the section of “Clinical significance of the MRI parameter” of brain feature namely “Left-Putamen-fMRI-ReHo- p10”.	As described in the section of “Reported changes of the brain region in CD” of brain feature namely “Ctx-Rh-Postcentral-fMRI-ReHo- p90”.	The decreased ReHo in CD patients may indicate the lower activation of this region and may be associated with sensorimotor and emotional dysfunctions.

The brain feature names highlighted in bold represent novel features that have not been previously documented in the literature

*p10* 10th percentile, *fMRI* functional MRI, *ctx* cortex, *ReHo* regional homogeneity, *DKI* diffusion kurtosis imaging, *KFA* kurtosis fractional anisotropy, *STD* standard deviation, *CBF* cerebral blood flow, *p90* 90th percentile, *vol* volume, *ALFF* amplitude of low-frequency fluctuations, *DKI* diffusion kurtosis imaging, *GA* geodesic anisotropy, *CD* Crohn’s disease, *HCs* healthy controls

* The changes in these MRI parameters have not been reported in CD

**Table 3:** The general importance of features in the brain radiomics model and the comprehensive brain-psychological-clinical model and the differences of these features between CD patients and HCs

Features	Mean SHAP value	Mean or median of features		
		HCs	CD patients	*p*-value
Neural radiomics model				
Left-Hippocampus R2Star p10	1.81	12.28 ± 1.29	11.26 ± 1.93	0.001
Left-Putamen fMRI ReHo p10	1.41	−1.00 ± 0.12	−0.91 ± 0.16	< 0.001
Ctx-Lh-Caudal-Anterior-Cingulate DKI KFA std	1.29	0.11 ± 0.01	0.13 ± 0.02	< 0.001
Ctx-Lh-Middle-Temporal CBF p90	1.13	131.01 [113.96, 142.40]	150.66 [127.77, 757.86]	< 0.001
Ctx-Rh-Postcentral fMRI ReHo p90	1.07	1.54 ± 0.25	1.31 ± 0.30	< 0.001
Ctx-Lh-Superior-Temporal vol	0.95	0.02 ± 0.001	0.01 ± 0.001	0.002
Brain-Stem fMRI ALFF p90	0.93	0.15 [0.02, 0.55]	0.65 [0.26, 1.07]	< 0.001
Left-Thalamus-Proper CBF std	0.78	14.32 [13.06, 16.79]	18.88 [15.73, 328.67]	< 0.001
Ctx-Lh-Cuneus fMRI ReHo std	0.66	0.78 ± 0.15	0.85 ± 0.17	0.02
Ctx-Rh-Superior-Frontal DKI GA std	0.49	0.17 ± 0.08	0.25 ± 0.17	0.003
Ctx-Rh-Precentral fMRI ALFF p10	0.47	0.36 [0.03, 0.55]	0.04 [−0.22, 0.27]	< 0.001
Ctx-Lh-Middle-Temporal vol	0.38	0.012 ± 0.001	0.011 ± 0.001	< 0.001
Ctx-Rh-Precentral fMRI ReHo mean	0.21	−0.49 ± 0.07	−0.55 ± 0.07	< 0.001
Clinical-psychological-neural model				
Rad-score	3.33	0.03 [0.002, 0.11]	0.10 [0.94, 0.10]	< 0.001
Alcohol	0.75	0 [0, 0]	0 [0, 1]	< 0.001
Education	0.55	6.00 [5.00, 6.00]	4.00 [4.00, 5.00]	< 0.001
S-score	0.40	29.35 ± 5.90	41.66 ± 10.26	< 0.001
BMI	0.34	22.68 ± 2.24	20.13 ± 3.34	< 0.001
T-score	0.27	31.37 ± 5.95	43.06 ± 9.95	< 0.001
PSS	0.25	29.24 ± 6.17	37.20 ± 7.91	< 0.001
BDI	0.25	3.70 ± 3.60	11.39 ± 9.46	< 0.001

Data are presented as mean ± standard deviation for data that follow a normal distribution, or as median (interquartile range) for data that do not follow a normal distribution

*p10* 10th percentile, *fMRI* functional MRI; *ctx* cortex, *ReHo* regional homogeneity, *DKI* diffusion kurtosis imaging, *KFA* kurtosis fractional anisotropy, *STD* standard deviation, *CBF* cerebral blood flow, *p90* 90th percentile, *vol* volume, *ALFF* amplitude of low-frequency fluctuations, *DKI* diffusion kurtosis imaging, *GA* geodesic anisotropy, *Rad-score* radiomics score, *S-score* state score of state-trait anxiety inventory, *T-score* trait score of state-trait anxiety inventory, *BMI* body mass index, *PSS* perceived stress scale, *BDI* Beck depression inventory, *CD* Crohn’s disease, *HCs* healthy controls

The RM effectively characterized the brain features of CD patients and successfully distinguished them from HCs, achieving an AUC of 0.988 (95% CI, 0.975–1.000) in training cohort and 0.914 (95% CI, 0.856–0.960) in validation cohort. In test cohort, RM still exhibited satisfactory performance (AUC = 0.956; 95% CI, 0.881–1.000) (Fig. 2d; Table 4). The optimal threshold determined for this RM was 0.56, indicating that when an individual’s rad-score exceeds 0.56, he/she is likely to exhibit the brain features associated with CD patients.

**Table 4:** The diagnostic performance of brain radiomics model and comprehensive brain-psychological-clinical model

	AUC	Sensitivity (%)	Specificity (%)	PPV (%)	NPV (%)	Delong *p*-value*
Brain radiomics model						
Training cohort	0.988 (0.975–1)	93.33	1	1	86.49	…
Validation cohort	0.914 (0.856–0.960)	88.00	78.12	90.41	73.53	…
Test cohort	0.956 (0.881–1)	91.18	92.86	96.88	81.25	…
Comprehensive model						
Training cohort	0.998 (0.993–1)	1	96.88	98.68	1	0.14
Validation cohort	0.994 (0.983–1)	96.00	96.88	98.63	91.18	0.01
Test cohort	0.985 (0.945–1)	1	92.86	97.14	1	0.10

Data in parentheses are 95% confidence interval

*AUC* area under the receiver operating characteristics curve, *NPV* negative predictive value, *PPV* positive predictive value

* *p*-value indicates the significance level of the comparison of AUCs between the brain radiomics model and comprehensive model in the corresponding cohort using DeLong test

### The performance of RM is comparable to that of a comprehensive brain-psychological-clinical model

**Fig. 3:**
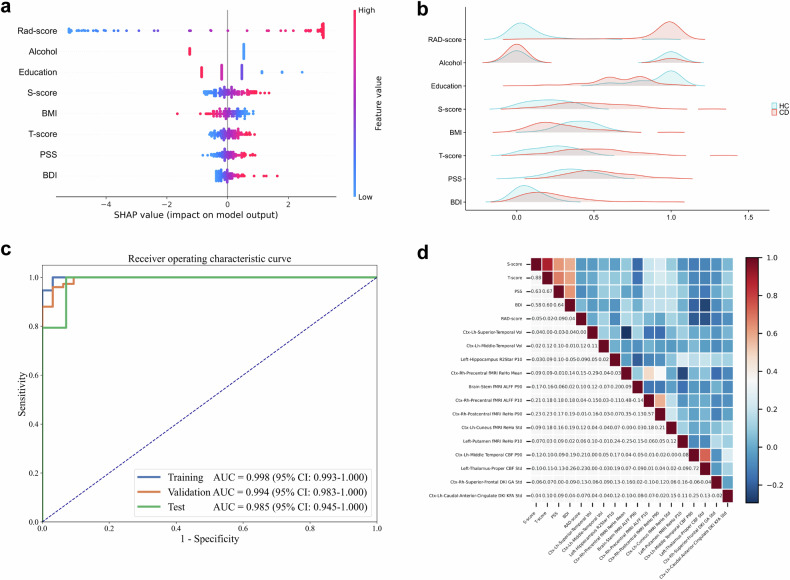
Development and assessment of a comprehensive brain-psychological-clinical model. **a** The SHAP summary plot of the comprehensive brain-psychological-clinical model (visual interpretation of the comprehensive model). The rad-score emerged as the most prominent determinant in the comprehensive model, surpassing all other clinical and psychological factors (see Table 3). SHAP plot illustrating the eight features used to develop brain-psychological-clinical model. The importance of the eight features is demonstrated in descending order. The horizontal location in the SHAP plot indicates whether the effect of that value is associated with a higher or lower prediction, and the color represents the level of that variable (high = red; low = blue) for that observation. Each dot represents a single sample. The height of the dots reflects the number of samples. **b** The ridge plots show the data distributions of these eight features in CD patients and HCs of the comprehensive model. Other detailed statistical information is shown in Table 3. **c** ROC curve of the comprehensive model for distinguishing CD patients from HCs. **d** Heatmap showing correlations among the 13 brain radiomics features, the rad-score, and the four psychological assessment scores. (rad-score, radiomics score; S, state score of the State-trait Anxiety Inventory, T, trait score of the State-trait Anxiety Inventory; BMI, body mass index; PSS, score on the Perceived Stress Scale; BDI, score on the Beck Depression Inventory; SHAP, Shapley Additive Explanations; CD, Crohn’s disease; HCs, healthy controls; p10, 10th percentile; fMRI, functional MRI; ctx, cortex; ReHo, regional homogeneity; DKI, diffusion kurtosis imaging; KFA, kurtosis fractional anisotropy; std, standard deviation; CBF, cerebral blood flow; p90, 90th percentile; vol, volume; ALFF, amplitude of low-frequency fluctuations; DKI, diffusion kurtosis imaging; GA, geodesic anisotropy; AUC, area under the ROC curve; CI, confidence interval)

The general importance of the eight features in the comprehensive model is shown in Table 3 and Fig. 3a. Specifically, the increase in the rad-score, S-score, T-score, PSS, and BDI had a positive impact and drove the prediction of the brain characteristics of CD patients. Among these features, rad-score had the most significant contribution to the comprehensive model, surpassing the importance of clinical and psychological factors, thereby further confirming its capability to characterize the brain features of CD. The differences in data distributions of these features between HCs and CD patients are illustrated in ridge plots (Fig. 3b). Specifically, the rad-score, S-score, T-score, PSS, and BDI were higher in CD patients than those of HCs. Conversely, CD patients demonstrated lower levels of education, BMI, and alcohol history when compared to HCs.

The comprehensive model also accurately distinguished CD patients from HCs, achieving AUCs of 0.998 (95% CI, 0.993–1.000), 0.994 (95% CI, 0.983–1.000), and 0.985 (95% CI, 0.945–1.000) in training, validation, and test cohorts, respectively (Fig. 3c). Notably, RM had comparable performance to that of the comprehensive model in training and test cohorts (DeLong’s test, both *p* > 0.05), suggesting that the single RM was able to characterize the brain features of CD patients.

Additionally, four brain features (Ctx-Rh-Precentral-fMRI-ALFF-P10, Ctx-Rh-Postcentral-fMRI-ReHo-P90, Ctx-Lh-Cuneus-fMRI-ReHo-Std, and Left-Thalamus-Proper-CBF-std) exhibited mild correlations with the S, T, and BDI scores (*r* = 0.10–0.26, all *p* < 0.05; Fig. 3d).

### The gut microbiota and blood metabolites differ between CD patients and HCs

**Fig. 4:**
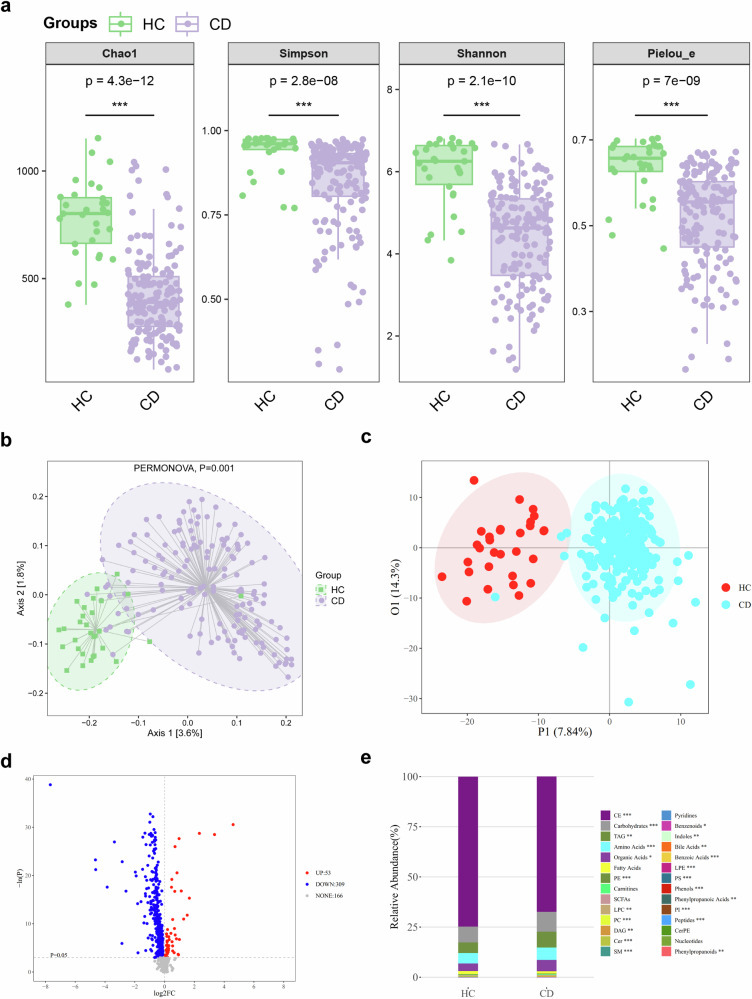
The differences in the gut microbiota and blood metabolites between CD patients and HCs. **a** Alpha diversity in gut microbiota between the CD patients and HCs. Compared to HCs, the alpha diversity of fecal samples was lower in CD patients (all *p* < 0.05). Alpha diversity represents within-sample microbial diversity. Chao1, Simpson, Shannon, and Pielou_e are four indices of alpha diversity, with specific meanings detailed in Supplementary Table 10. Higher values of these indices indicate better alpha diversity and greater benefits to individuals. **b** Comparison of beta diversity in the gut microbiota between the CD patients and HCs. The beta diversity of fecal samples differed between HCs and CD patients (PERMANOVA, *p* = 0.001). Beta diversity represents between-sample microbial diversity. PCA plot of microbiome beta diversity showing the different clusters of CD patients (purple) and HCs (green). Squares and circles represent samples of HCs and CD patients. **c** The OPLS-DA score plot demonstrates distinct differences in blood metabolites between HCs and CD patients. OPLS-DA score plot showing the different clusters of CD patients (blue) and HCs (red). Dots in red and blue represent samples of HCs and CD patients. **d** Volcano plot of the univariate analysis comparing CD patients and HCs. The red dots in the right top region represent higher levels of differentially abundant blood metabolites in CD patients than in HCs, while the blue dots in the left top region indicate lower levels of differentially abundant blood metabolites in CD patients than in HCs. **e** Relative abundance of each blood metabolite class between CD patients and HCs. On the right side, the specific names of the detected 28 metabolites and the significance of the inter-group differences are shown. *p < 0.05; **p < 0.005; ***p < 0.001. (CE, cholesteryl ester; TAG, triacylglycerol; PE, phosphatidylethanolamine; SCFAs, short-chain fatty acids; LPC, lysophosphatidylcholine; PC, phosphatidylcholine; DAG, diacylglycerol; Cer, ceramide; SM, sphingomyelin; LPE, lysophosphatidylethanolamine; PS, phosphatidylserine; PI, phosphatidylinositol; CerPE, ceramide phosphoethanolamines; CD, Crohn’s disease; HCs, healthy controls)

Alpha diversity was significantly lower in CD patients than in HCs (Fig. 4a). Beta diversity also differed significantly between the groups (Fig. 4b). In LEfSe differential analysis of the gut microbiota, 24 genera were found to be enriched in CD patients, including *Shigella*, *Ruminococcus*, *Lactobacillus*, *Veillonella*, *Clostridium*, and *Enterococcus* (Supplementary Table 6).

Five hundred and twenty-eight metabolites were identified by metabolomic profiling. The blood metabolites were significantly different between the two groups (Fig. 4c). A volcano plot based on the univariate analysis showed the concentration trend of differentially abundant metabolites (Fig. 4d). The relative abundance of each metabolite class in the CD and HC groups was observed (Fig. 4e). Cholesterylester (CE), carbohydrate, and triacylglycerol (TAG) were the most abundant metabolite classes that exhibited significant differences. The percentages of these three metabolites in CD patients and HCs were 67.37% vs. 74.71% for CE, 9.87% vs. 8.00% for carbohydrate, and 7.92% vs. 5.28% for TAG, respectively.

### The brain features correlate with CD-enriched microbiota and blood metabolites

**Fig. 5:**
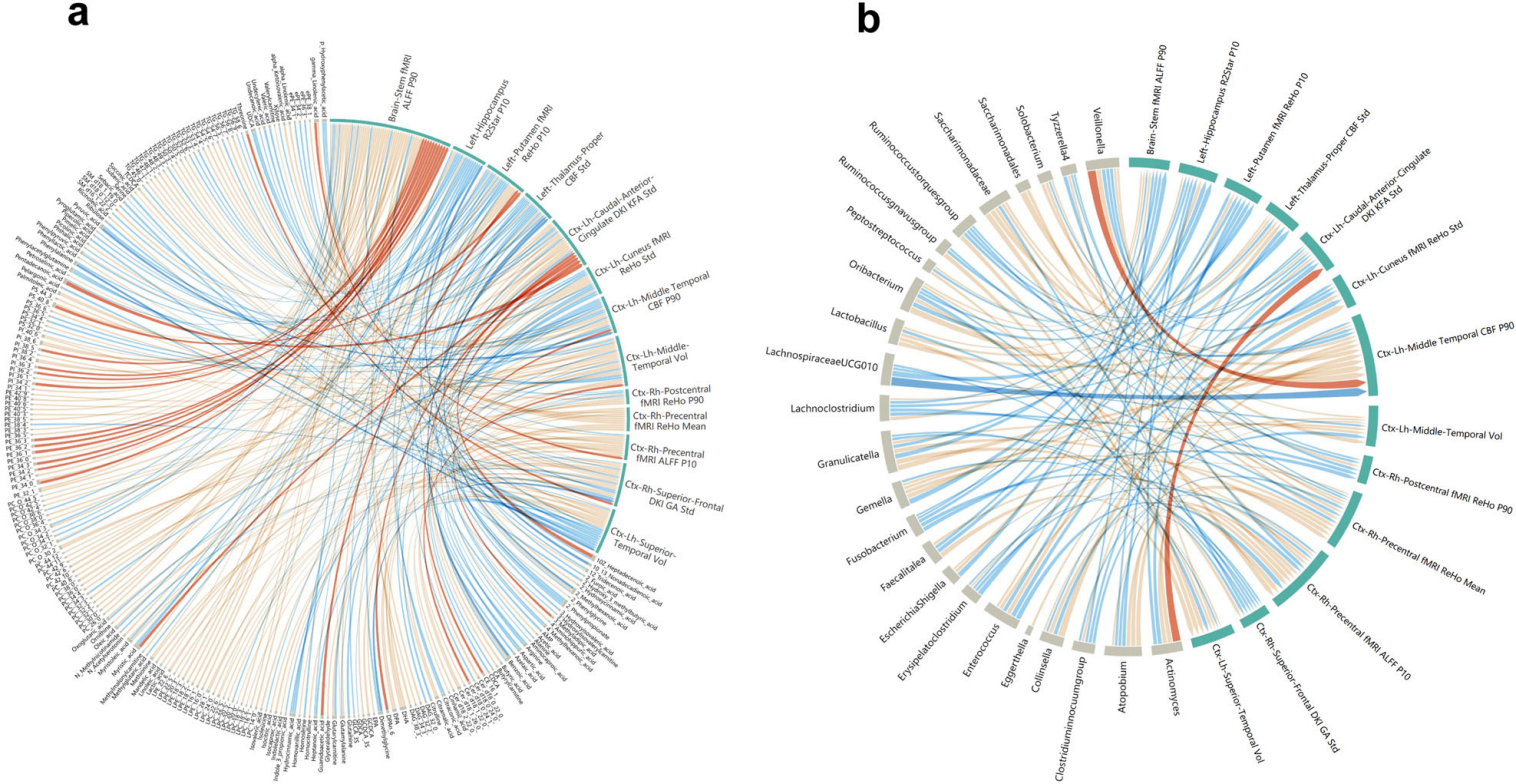
Chord diagram displaying the relationships among blood metabolites, brain radiomics features, and CD-enriched genera. **a** The chord diagram illustrates the relationships between 137 blood metabolites and thirteen brain features of CD patients, as determined through linear regression analysis adjusting for age, sex, and body mass index. The width of each arc on the circumference reflects how frequently a variable shows significant correlations with others, while also considering the correlation coefficient. The latter is also shown through variations in string thickness and color intensity; thicker strings indicate stronger correlations, while redder colors indicate positive correlations and bluer colors indicate negative correlations (see Supplementary Table 7). **b** The chord diagram illustrates the relationships between 24 CD-enriched genera and 13 brain features of CD patients determined through linear regression analysis adjusting for age, sex, and body mass index (see Supplementary Table 9). (AMP, adenosine monophosphate; CA, cholic acid; CDCA, chenodeoxycholic acid; CE, cholesteryl ester; CER, ceramide; DAG, diacylglycerol; DHA, docosahexaenoic acid; DPA, docosapentaenoic acid; EPA, eicosapentaenoic acid; GCDCA_s, glycochenodeoxycholic acid sulfate; LPC, lysophosphatidylcholine; LPE, lysophosphatidylethanolamine; PC, phosphatidylcholine; PE, phosphatidylethanolamine; PI, phosphatidylinositol; PS, phosphatidylserine; UDCA, ursodeoxycholic acid; SM, sphingomyelin; TAG, triacylglycerol; bUDCA, 3β-ursodeoxycholic acid; DAG, diacylglycerol; SM, sphingomyelin; ctx, cortex; vol, volume; p10, 10th percentile; fMRI, functional MRI; ReHo, regional homogeneity; ALFF, amplitude of low-frequency fluctuations; p90, 90th percentile; std, standard deviation; p10, 10th percentile; CBF, cerebral blood flow; DKI, diffusion kurtosis imaging; GA, geodesic anisotropy; KFA, kurtosis fractional anisotropy)

The correlations among 13 brain MRI features, 24 CD-enriched microbiota, and 528 blood metabolites were analyzed in a cohort of 55 CD patients with complete multiomics data. Age, sex, and BMI were corrected as covariates to avoid potential confounding effects when we conducted pairwise regression analysis. Only the variables significant in the pairwise regression analysis (*p* < 0.05) are shown in chord diagram. Significant correlations were detected between the 13 brain radiomics features and 137 blood metabolites (Fig. 5a; Supplementary Table 7), and between nine bacterial genera and 122 blood metabolites (Supplementary Fig 3; Supplementary Table 8); additionally, 24 bacterial genera were correlated with 13 brain features (Fig. 5b; Supplementary Table 9). Notably, TAG, phosphatidylcholine (PC), and phosphatidylethanolamine (PE) exhibited significant correlations with both brain features (Fig. 5a) and CD-enriched microbiota (Supplementary Fig. 3). Moderate correlations were found between *LachnospiraceaeUCG010* and Ctx-Lh-Middle-Temporal CBF-p90 (*r* = −0.43) and between *Veillonella* and Ctx-Lh-Middle-Temporal CBF-p90 (*r* = 0.41). In addition, we also found mild correlations across multiple sets of data (|*r*| =0.20–0.39). Among them, we focused on the association between CD pathogens (such as *Enterococcus* and *Collinsella*) and brain features. We found that the more obvious correlations were found between *Enterococcus* and Ctx-Rh-Precentral fMRI ALFF p10 (*r* = −0.26), between *Enterococcus* and Ctx-Lh-Caudal-Anterior-Cingulate-DKI-KFA-std (*r* = −0.25), between *Collinsella* and Ctx-Lh-Cuneus-fMRI-ReHo-std (*r* = 0.35) (Fig. 5b).

**Fig. 6:**
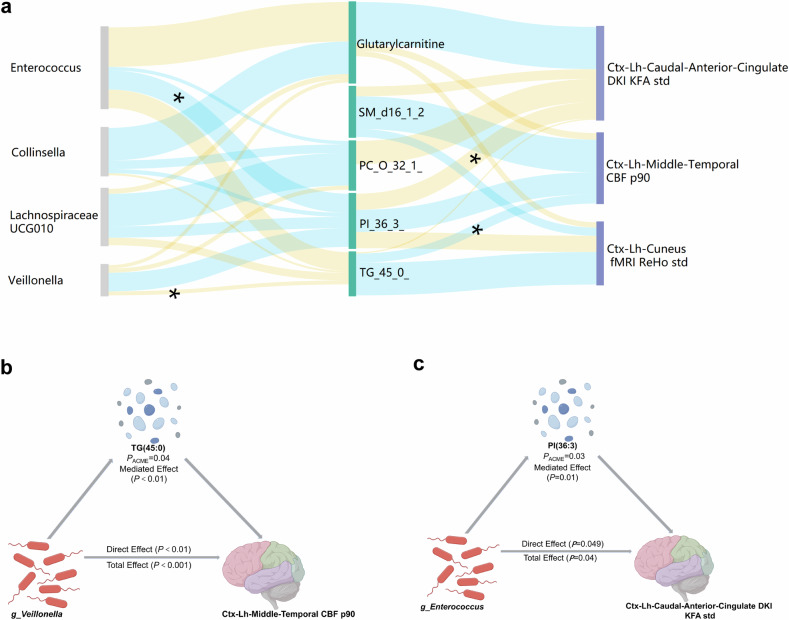
Putative links among alterations in the gut microbiota, blood metabolites, and the brain of CD patients. **a** Sankey plot indicating microbial genera contributing to brain radiomics features in patients with CD, mediated by blood metabolites. All *p*-values of average causal mediation effects are either below 0.05 (*p < 0.05) or approaching it (*p* = 0.052–0.066). The height of the column reflects the sum of the related features and the absolute value of its correlation coefficient. The width of each band represents the correlation coefficient value, and the wider the band is, the greater the correlation coefficient. Yellow indicates a positive correlation, and blue indicates a negative correlation. **b** Illustration of the association between g_Veillonella and brain features (i.e., Ctx-Lh-Middle-Temporal CBF p90) mediated by TAG (45:0) in CD patients (*p*_ACME_ = 0.038). **c** Illustration of the association between g_Enterococcus and brain features. (Ctx-Lh-Caudal-Anterior-Cingulate-DKI-KFA std) in CD patients (*p*_ACME_ = 0.026). (ACME, average causal mediation effects; TAG, triacylglycerol; g, genus; ctx, cortex; lh, left head; CBF, cerebral blood flow; p90, 90th percentile; PI, phosphatidylinositol; g, genus; DKI, diffusion kurtosis imaging; KFA, kurtosis fractional anisotropy; std, standard deviation)

Subsequently, we conducted a mediation analysis to investigate the potential effects of gut microbiota and metabolites on brain MRI features (Fig. 6a). We identified two significant pathways (both *p* < 0.05; Fig. 6b, c), while other pathways exhibited approaching statistical significance (*p* = 0.052–0.066), indicating potential causal links from the gut microbiota to blood metabolites and then to brain alterations. In these two significant pathways, the alteration in cerebral blood flow (CBF) in the left middle temporal cortex was caused by an elevated blood TAG level (45:0) due to *Veillonella* metabolism (*p*_ACME_ < 0.05) (Fig. 6b), while the enriched genus *Enterococcus* influenced the microstructure of the left caudal anterior cingulate cortex (detectable by kurtosis fractional anisotropy (KFA), from diffusion kurtosis imaging (DKI)) through blood phosphatidylinositol levels (*p*_ACME_ < 0.05) (Fig. 6c).

## Discussion

Among the thirteen brain features identified in our study, imaging parameters or specific brain regions of the eight brain features have been previously reported in studies on CD, supporting the reliability of our findings. The remaining five brain features, including lower R2star values in the left hippocampus, higher KFA (from DKI) in the left caudal anterior cingulate cortex, increased geodesic anisotropy (GA; from DKI) in the right superior-frontal cortex, and enhanced CBF in the left middle temporal cortex and left thalamus, have not yet been documented in CD patients. The identification of these five novel brain features may offer additional insights into the brain characteristics of CD patients. Subsequently, we developed an RM using these multiparametric brain MRI for accurate characterization of brain features of CD patients. The multiomics data revealed significant and intricate relationships between brain MRI features, gut microbiota, and blood metabolites, thereby supporting the validity of this RM.

Brain MRI can reveal neuropsychological changes in CD patients [48]. In our RM, R2star value in the left hippocampus presented the highest mean SHAP value, indicating that it was the most distinguishing factor among the 13 brain features of CD patients. A previous animal study indicated that experimental colitis affects hippocampal neurogenesis and innate immune cell responses [49]. Our study reported the alteration of R2star values in the left hippocampus of CD patients; we hypothesized that it may detect these cell responses by reflecting iron deposition in this region. Additionally, we also identified five previously unreported brain features in CD patients (interpreted in detail in Table 2), thereby providing novel insights into CD neurophenotype.

Another six brain features in our study, including Left-Putamen-fMRI-ReHo-p10, Ctx-Rh-Postcentral-fMRI-ReHo-p90, Brain-Stem-fMRI-ALFF-p90, Ctx-Lh-Cuneus-fMRI-ReHo-std, Ctx-Rh-Precentral-fMRI-ALFF-p10, and Ctx-Rh-Precentral-fMRI-ReHo-mean, were consistent or partially overlapped with previous studies in terms of the same brain region and/or fMRI parameters that reported for CD patients [12, 50] (detailed in Table 2). Among them, the changes of Ctx-Rh-Postcentral-fMRI-ReHo-p90 in our study were consistent with the findings reported in a previous study, where this feature value of HCs was higher than that of CD [12]. These brain regions are primarily associated with emotion, pain, or cognitive functions [12, 50]. Among them, the putamen may serve as a pathway through which the gut microbiota can enhance visceral hypersensitivity [51]. In our study, ReHo alterations in the putamen were associated with levels of aspartic acid (related to brain neurotransmitter synthesis [52]) and glycochenodeoxycholic acid 3-sulfate (GCDCA_3S, related to intestinal health [53]), suggesting that this variable may reflect disruption of the gut-brain axis. Additionally, the postcentral gyrus plays a crucial role in perceiving somatic sensations, including pressure and pain [54, 55], which aligns with our findings that PSS and pain scores were higher in CD patients than in HCs. Overall, our RM offers a comprehensive picture of the neural alterations in CD patients, thereby clarifying the neurophenotype of CD.

The investigation into the underlying correlation of neural alterations with gut will enhance our understanding of the neurophenotypes of CD patients. The gut-brain axis provides a framework for elucidating the interplay between gut and brain alterations [56]. In microbiome analysis, we found that alpha diversity in CD patients was lower compared to HC, and beta diversity also showed significant differences from HC, consistent with previous literature [57]. Most of these genera identified by LEfSe analysis, such as *Shigella*, *Ruminococcus*, and *Veillonella*, were reported to be enriched in previous CD studies [58, 59], suggesting that our findings were reliable. Moreover, we found that the relative abundance of four genera, *Veillonella*, *LachnospiraceaeUCG010*, *Collinsella*, and *Enterococcus*, was significantly correlated with both brain MRI features and numerous blood metabolites. The microbial metabolite pathway has been demonstrated to be a potential correlation by which the microbiota can affect brain function [60]. We conducted association and mediation analyses and identified two significant pathways linking features of the gut microbiota, blood metabolites, and the brain in CD patients. Among these pathways, the “genus *Veillonella*”-“TAG (45:0)”-“CBF of the left middle temporal cortex” axis was prominent. The increased abundance of the genus *Veillonella* contributes to CD pathogenesis [59, 61], while excess TAG levels can cause tissue inflammation [62, 63]The interplay between *Veillonella* abundance and TAG (45:0) may increase the CBF in the left middle temporal cortex, thereby inducing distinctive brain features specific to CD patients. Similarly, the genus *Enterococcus* and blood levels of phosphatidylinositol, which has implications for inflammation regulation [64], influenced the microstructure of the left caudal anterior cingulate cortex according to KFA values derived from DKI. All these findings support the hypothesis that alterations in the gut microbiota and blood metabolites are associated with brain changes in CD patients through gut-brain axis, thereby validating the alterations in brain MRI. Clinical and preclinical studies have substantiated the efficacy of brain-gut axis-based emotional therapy in ameliorating intestinal lesions and clinical markers [65–67].

This study had some limitations. First, this was a single-center study. However, the participants were living in various regions in China, and the data were collected over nearly three years. Hence, our RM had good representativeness. Second, the number of participants with complete multiomics data was limited, and not all the included participants underwent brain MRI examination. In future studies, a larger sample size with a complete set of data could confirm our findings. Finally, the potential pathways identified in our study require further validation. While such validation is beyond the scope of this study, future research is warranted to address this.

In conclusion, the brain function and structure of CD patients exhibited subtle yet distinct differences from those of HCs, the multiparameter MRI-based RM we developed can characterize the neural alterations of CD patients. The validity of this RM is further supported by presenting biologically plausible evidence using multiomics data. This RM provides additional information that can enhance understanding of the CD neurophenotype and serve as a biomarker for better management of CD patients.

## Supplementary information

ELECTRONIC SUPPLEMENTARY MATERIAL

## Data Availability

The datasets used and analyzed during the current study are available from the corresponding author upon reasonable request.

## Abbreviations

ALFF: Amplitude of low-frequency fluctuations
AUC: Area under ROC curve
BDI: Beck Depression Inventory
BMI: Body mass index
CBF: Cerebral blood flow
CD: Crohn’s disease
CI: Confidence interval
Ctx: Cortex
DKI: Diffusion kurtosis imaging
fMRI: Functional MRI
GA: Geodesic anisotropy
HCs: Healthy controls
KFA: Kurtosis fractional anisotropy
LR: Logistic regression
PSS: Perceived Stress Scale
Rad-score: Radiomics Score
ReHo: Regional homogeneity
RM: Radiomics model
ROC: Receiver operating characteristic
SHAP: Shapley Additive Explanations
S-score: State Score of State-trait Anxiety Inventory
T1WI: T1-weighted imaging
TAG: Triacylglycerol
T-score: Trait Score of State-trait Anxiety Inventory
