# Supplementary material for: Identification of neural alterations in patients with Crohn’s disease with a novel multiparametric brain MRI-based radiomics model
Source: Insights Imaging. 2024 Nov 29;15:289. doi: 10.1186/s13244-024-01859-6 (PMC11607295; doi:10.1186/s13244-024-01859-6)

Identification of neural alterations in patients with Crohn's disease with a novel multiparametric brain MRI-based radiomics model

ELECTRONIC SUPPLEMENTARY MATERIAL

Materials and Methods

Supplementary Materials

1. Brain MRI data preprocessing……………………………………………………….....4

2. Development and validation of brain radiomics model…………..…………………..5

3. Development of comprehensive brain-psychological-clinical model ……………7

4. Detailed information of psychological questionnaire…………………………………8

5. Fecal 16S rRNA sequencing and blood metabolomics profiling……………………11

References …………………………………………………………………….…………..14

Supplementary Tables

Supplementary Table. 1 The protocol parameters of multiparameter MRI sequences…...15

Supplementary Table. 2 Perceived Stress Scale……………………………………….…….16

Supplementary Table. 3 State-Trait Anxiety Inventory (State score)……………….….…17

Supplementary Table. 4 State-Trait Anxiety Inventory (Trait score)…………………..…..18

Supplementary Table. 5 Beck's Depression Inventory…………………………..…………19

Supplementary Table. 6 Relative abundance of the 24 CD-enriched bacteria genera…..22

Supplementary Table. 7 Pearson correlation coefficients between brain radiomics features and blood metabolites…………………………………………………………………….…23

Supplementary Table. 8 Pearson correlation coefficients between CD-enriched bacteria genera and blood metabolites……………………….……………………………………….29

Supplementary Table. 9 Pearson correlation coefficients between CD-enriched bacteria genera and brain radiomics features…………………………..…………….…………….34

Supplementary Table. 10 Definition and index introduction of α and β diversity………...36

Supplementary Figures

Supplementary Figure. 1 Flowchart shows participants recruitment process……………....38

Supplementary Figure. 2 Flow-chart of the feature selections steps………………………..39

Supplementary Figure. 3 Chord diagram displaying the relationships among blood metabolites and CD-enriched genera………… ……………..……………………………..40

Supplementary Materials

Supplementary Materials 1: *Brain MRI data preprocessing*

Cerebral Blood Flow (CBF) images were obtained through post-processing of ASL (Arterial Spin Labeling) images using the "explore ASL" software[1]. Quantitative Susceptibility Mapping (QSM) images were reconstructed using the Morphology Enabled Dipole Inversion (MEDI) method from the MEDI Toolbox[2], and R2star mapping images were acquired from the scans directly, both of which can be used to measure iron deposition. Amplitude of Low-Frequency Fluctuations (ALFF) images and Regional Homogeneity (ReHo) images were the physiological variables extracted from Blood-Oxygen-Level-Dependent (BOLD) functional MRI (fMRI).

Diffusion-weighted imaging (DWI) images was utilized to compute various diffusion models. The Diffusion Imaging in Python (DIPY) open-source toolkit[3] was employed to calculate Diffusion Tensor Imaging (DTI) metrics, including Fractional Anisotropy (FA), Mean Diffusivity (MD), Axial Diffusivity (AD), and Radial Diffusivity (RD). Additionally, Diffusion Kurtosis Imaging (DKI) metrics, including FA, MD, AD, RD, Mean Kurtosis (MK), Axial Kurtosis (AK), Radial Kurtosis (RK), Geodesic Anisotropy (GA) and Kurtosis Fractional Anisotropy (KFA) were computed. Moreover, Neurite Orientation Dispersion and Density Imaging (NODDI) metrics, encompassing Intra-cellular Volume Fraction (ICVF), Isotropic Volume Fraction (ISOVF), and Orientation Dispersion Index (ODI), were also calculated. Lastly, Mean Apparent Propagator (MAP) were computed, which included Mean Squared Displacement (MSD), Non-Gaussian (NG), Axial Non-Gaussian (NGAx), Radial Non-

Gaussian (NGRad), Q-space Inverse Variance (QIV), Return-To-Axis Probability (RTAP), Return-To-Origin Probability (RTOP), and Return-To-Plane Probability (RTPP).

Supplementary Materials 2: *Development and validation of brain radiomics model*

Before modeling, the training cohort was balanced using an up-sampling technique to ensure an equal proportion of positive and negative samples in the dataset. The first-order radiomics features were standardized using the Z-SCORE algorithm.

To reduce the dimensionality and select the best subset of features, a three-step strategy were used. Initially, 2755 (29 parameter maps×95 brain regions) single-parameter-single-brain-region logistic regression (LR) models were constructed to predict the respective tasks, using features selected from five first-order features by the recursive feature elimination algorithm (RFE), respectively. Additionally, 95 univariate LR models were performed with brain regions vol. The performance of 2850 models was evaluated using the area under the receiver operating characteristic (ROC) curve (AUC). Secondly, twenty-nine single-parameter-multi-brain-region LR models and a multi-brain-region vol model were constructed by RFE selecting and combining the features used in single-parameter-single-brain-region model that was deemed eligible for inclusion into the single-parameter-multi-brain-region model was the one with an AUC surpassing a certain threshold (set at 0.6). For each single-parameter-multi-brain-region model, features ranging from 12 to 152 were included and ultimately 1 to 15 features were selected by the RFE. Thirdly, the RFE algorithm was then applied again to select the ultimate 13 features from the 347 candidates (combining of all single-parameter-multi-brain-region models in step 2; **Supplementary Fig.2**) to develop the multi-parameter-multi-brain-region model (**Fig.2A**). To ensure the robustness and

generalization of the multi-parameter model, five-fold cross-validation was performed on the training set to determine the final feature set. The radiomics score (rad-score) for each participant was calculated according to the final multi-parameter-multi-brain-region model. The construction of RM was implemented with the open-source software FeatureExplore.

The final RM was: $sigmoid((\sum_{k=0}^{n} \frac{(FeatureK - interception)}{slope} * Coef) + 0.3343)$

where "*FeatureK*" is value of brain feature which finally selected by RM, "$interception$", "$Coef$", and "$slope$" are values generated by RM for each brain feature, 0.3343 is bias of linear regression.

Supplementary Materials 3: *Development of comprehensive brain-psychological-clinical model*

A multivariate LR analysis was employed to evaluate the combined effects of rad-score, clinical variates, and psychological scales on the target tasks, thereby constructing a comprehensive brain-psychological-clinical model for comparison with the single RM (using DeLong's test).

The formula was: $sigmoid((\sum_{k=0}^{n}\frac{(FeatureK - interception)}{slope} * Coef) + 0.4194)$.

The "FeatureK" comprises eight variables, namely RAD-score, perceived stress scale (PSS), beck depression inventory (BDI), trait score of state-trait anxiety inventory (T-score), state score of state-trait anxiety inventory (S-score), body mass index (BMI), Alcohol, and Education. These variables were ultimately selected by significance testing. The values for each variable, namely "interception," "Coef," and "slope," were generated by LR, 0.4194 is bias of linear regression.

Supplementary Materials 4: ***Detailed information of psychological questionnaire***

Prior to the commencement of the project, the radiologists responsible for assessing psychological scales underwent professional training in our hospital's neuropsychology department to acquaint themselves with the evaluation process of these psychological scales. When administering the psychological scale assessment to patients, they will provide guidance on completing the items in accordance with the scale guidelines.

1) **State-trait anxiety inventory (STAI)**

Purpose: To measure via self-report the presence and severity of current symptoms of anxiety and a generalized propensity to be anxious.

Content: There are 2 subscales within this measure. First, the State Anxiety Scale (S-Anxiety) evaluates the current state of anxiety, asking how respondents feel “right now” using items that measure subjective feelings of apprehension, tension, nervousness, worry, and activation/arousal of the autonomic nervous system. The Trait Anxiety Scale (T-Anxiety) evaluates relatively stable aspects of “anxiety proneness” including general states of calmness, confidence, and security.

Method of administration: Paper and pencil administration. This is a self-report questionnaire that can be administered in an individual format. Specific instructions are provided for each of the S-Anxiety and T-Anxiety subscales.

Respondent burden: For adults, this measure requires ~10 minutes to complete.

Scoring: Item scores are added to obtain subtest total scores. Scoring should be reversed for anxiety-absent items (19 items of the total 40).

Score interpretation: Range of scores for each subtest is 20–80, the higher score indicating

greater anxiety. A cut point of 39–40 has been suggested to detect clinically significant symptoms for the S-Anxiety scale; however, other studies have suggested a higher cut score of 54–55 for older adults. Normative values are available in the manual for adults, college students, and psychiatric samples[4].

2) Perceived stress Scale (PSS)

Purpose: Perceived Stress Scale, a 14-item measure of the degree to which situations in one's life are appraised as stressful. PSS items were designed to tap the degree to which respondents found their lives unpredictable, uncontrollable, and overloading. These three issues have been repeatedly found to be central components of the experience of stress.

Method of administration: The questions in this scale ask you about your feelings and thoughts during the last month. In each case, you will be asked to indicate how often you felt or thought a certain way. Although some of the questions are similar, there are differences between them and you should treat each one as a separate question. The best approach is to answer each question fairly quickly. That is, don't try to count up the number of times you felt a particular way, but rather indicate the alternative that seems like a reasonable estimate.

Score interpretation: Total scores can range from 0 to 56 with higher scores indicating greater stress[5].

3) Beck depression inventory (BDI)

Purpose & Content: The BDI was derived from clinical observations about the attitudes and symptoms displayed frequently by depressed psychiatric patients and infrequently by nondepressed psychiatric patients (Beck et al., 1961). The clinical observations were consolidated systematically into 21 symptoms and attitudes which could be rated from 0 to 3

in terms of intensity. The items were chosen to assess the intensity of depression and were not selected to reflect a particular theory of depression.

Method of administration: Although the BDI was initially designed to be administered by trained interviewers, it is most often self-administered. When self-administered, the instrument generally takes 5-10 minutes to complete and is scored by summing the ratings given to each of the 21 items.

Score interpretation: The Center for Cognitive Therapy has distributed the following guidelines for BDI cut-off scores with patients diagnosed as having an affective disorder: none or minimal depression is < 10; mild to moderate depression is 10-18; moderate to severe depression is 19-29; and severe depression is 30-63[6].

Supplementary Materials 5: ***Fecal 16S rRNA sequencing and blood metabolomics profiling***

The participants provided fecal and/or serum samples in the hospital. The feces were collected in collection cups and immediately frozen at −80 °C until analysis. Blood samples were drawn after overnight fasting, sent directly to the laboratory for obtaining the serum, and also frozen at −80 °C until analysis.

A total of 152 patients with CD as well as 30 HCs provided fecal samples for the purpose of conducting amplicon sequencing on the 16S rRNA gene. Total genomic DNA samples were extracted using the OMEGA Soil DNA Kit (M5635-02) (Omega Bio-Tek, Norcross, GA, USA), according to the manufacturer's instructions, and subsequently stored at -20 °C for further analysis. The quantity and quality of extracted DNAs were measured using a NanoDrop NC2000 spectrophotometer (Thermo Fisher Scientific, Waltham, MA, USA) and agarose gel electrophoresis, respectively.

The V3-V4 region of bacterial 16S rRNA genes was amplified by Polymerase Chain Reaction (PCR) using the forward primer 338F (5'-ACTCCTACGGGAGGCAGCA-3') and the reverse primer 806R (5'-GGACTACHVGGGTWTCTAAT-3'). Sample-specific 7-bp barcodes were incorporated into the primers for multiplex sequencing. The PCR components contained 5 μl of buffer (5×), 0.25 μl of Fast pfu DNA Polymerase (5U/μl), 2 μl (2.5 mM) of dNTPs, 1 μl (10 uM) of each forward and reverse primer, 2 μl of DNA Template, and 8.75 μl of ddH2O. Thermal cycling consisted of initial denaturation at 98 °C for 2 min, followed by 25 cycles consisting of denaturation at 98 °C for 15 s, annealing at 55 °C for 30 s, and extension at 72 °C for 30 s, with a final extension of 5 min at 72 °C. PCR amplicons were purified with

Vazyme VAHTSTM DNA Clean Beads (Vazyme, Nanjing, China) and quantified using the Quant-iT PicoGreen dsDNA Assay Kit (Invitrogen, Carlsbad, CA, USA). After the individual quantification step, amplicons were pooled in equal amounts, and pair-end 2×250 bp sequencing was performed using the Illlumina NovaSeq platform with NovaSeq 6000 SP Reagent Kit (500 cycles) at Shanghai Metabo-Profile Biotechnology Co., Ltd (Shanghai, China).

Microbiome bioinformatics analysis was conducted using QIIME2 2019.4[7] based on the official tutorials (https://docs.qiime2.org/2019.4/tutorials/). Briefly, raw sequence data were demultiplexed using the demux plugin, followed by primers cutting with the cutadapt plugin[8]. Sequences were then quality filtered, denoised, merged and chimera removed using the DADA2 plugin[9]. Non-singleton amplicon sequence variants (ASVs) were aligned with mafft[10] and used to construct a phylogeny with fasttree2[11]. Taxonomy was assigned to ASVs using the classify-sklearn naïve Bayes taxonomy classifier in feature-classifier plugin[12] against the SILVA Release 132 Database[13].

Blood samples were collected from all 260 participants for the purpose of targeted metabolomics profiling. The targeted metabolomics of tissue samples were performed by Metabo-Profile (Shanghai, China). The sample preparation procedures were according to the previously published methods with minor modifications[14]. Briefly, 20 μL of serum was transferred to a 96-well plate. Subcequently, 120 μL of methanol containing internal standard to extract the metabolites. The resulting supernatants were subjected to derivatization with 3-nitrophenylhydrazine (3-NPH) and N-(3-(dimethylamino)propyl)-N′-ethylcarbodiimide (EDC)·HCl (Sigma-Aldrich, St. Louis, MO, USA). Subsequently, the derivatized samples were

analyzed by ultraperformance liquid chromatography coupled to tandem mass spectrometry (UPLC-MS/MS) system (ACQUITY UPLC Xevo TQ-S, Waters Corp., Milford, MA, USA). All of the standards were obtained from Sigma-Aldrich (St. Louis, MO, USA), Steraloids Inc. (Newport, RI, USA) and TRC Chemicals (Toronto, ON, Canada). The quality control samples were prepared following the same procedures as the test samples and were injected at regular intervals to ensure the instrument system stability. The raw data files generated by UPLC-MS/MS were processed using the Targeted Metabolome Batch Quantification (TMBQ) software (v1.0, HMI, Shenzhen, Guangdong, China) to perform peak integration, calibration, and quantitation for each metabolite. The self-developed platform iMAP (v1.0, Metabo-Profile, Shanghai, China) was used for statistical analysis.

References

1 Mutsaerts HJMM, Petr J, Groot P et al (2020) ExploreASL: An image processing pipeline for multi-center ASL perfusion MRI studies. NeuroImage 219:117031

2 Emmerich J, Bachert P, Ladd ME, Straub S (2021) A novel phantom with dia- and paramagnetic substructure for quantitative susceptibility mapping and relaxometry. Physica Medica : PM : an International Journal Devoted To the Applications of Physics To Medicine and Biology : Official Journal of the Italian Association of Biomedical Physics (AIFB) 88:278-284

3 Jordan K, Morin O, Wahl M et al (2019) An Open-Source Tool for Anisotropic Radiation Therapy Planning in Neuro-oncology Using DW-MRI Tractography. Frontiers In Oncology 9:810

4 Julian LJ (2011) Measures of anxiety: State-Trait Anxiety Inventory (STAI), Beck Anxiety Inventory (BAI), and Hospital Anxiety and Depression Scale-Anxiety (HADS-A). Arthritis Care & Research 63 Suppl 11:S467-S472

5 Cohen S, Kamarck T, Mermelstein R (1983) A global measure of perceived stress. Journal of Health and Social Behavior 24:385-396

6 Beck AT, Steer RA, Carbin MG (1988) Psychometric properties of the Beck Depression Inventory: Twenty-five years of evaluation. Clinical Psychology Review 8:77-100

7 Bolyen E, Rideout JR, Dillon MR et al (2019) Reproducible, interactive, scalable and extensible microbiome data science using QIIME 2. Nature Biotechnology 37:852-857

8 Kechin A, Boyarskikh U, Kel A, Filipenko M (2017) cutPrimers: A New Tool for Accurate Cutting of Primers from Reads of Targeted Next Generation Sequencing. Journal of Computational Biology : a Journal of Computational Molecular Cell Biology 24:1138-1143

9 Callahan BJ, McMurdie PJ, Rosen MJ, Han AW, Johnson AJA, Holmes SP (2016) DADA2: High-resolution sample inference from Illumina amplicon data. Nature Methods 13:581-583

10 Katoh K, Misawa K, Kuma K-i, Miyata T (2002) MAFFT: a novel method for rapid multiple sequence alignment based on fast Fourier transform. Nucleic Acids Research 30:3059-3066

11 Price MN, Dehal PS, Arkin AP (2009) FastTree: computing large minimum evolution trees with profiles instead of a distance matrix. Molecular Biology and Evolution 26:1641-1650

12 Bokulich NA, Kaehler BD, Rideout JR et al (2018) Optimizing taxonomic classification of marker-gene amplicon sequences with QIIME 2's q2-feature-classifier plugin. Microbiome 6:90

13 Kõljalg U, Nilsson RH, Abarenkov K et al (2013) Towards a unified paradigm for sequence-based identification of fungi. Molecular Ecology 22:5271-5277

14 Xie G, Wang L, Chen T et al (2021) A Metabolite Array Technology for Precision Medicine. Analytical Chemistry 93:5709-5717

Supplementary Table 1 The protocol parameters of multiparameter MRI sequences

Sequence	Slice orientation	FoV read(mm)	FoV phase (%)	Matrix	TR (ms)	TE (ms)	TI (ms)	Slice thickness(mm)	Averages
T1W	Sag	256	100	320x320	1800	2.54	911	0.80	1
BOLD	Axial	211	100	88x88	1500	30	…	2.40	1
DSI	Axial	220	100	110x110	3700	72	…	2	1
QSM	Axial	220	78.10	282x448	55	6.72	…	2	1
ASL	Axial	256	100	63x64	4000	15.72	…	4	1

Note. —Sag, sagittal; FoV, field of view; TR, repetition time; TE, echo time; TI, inversion time; T1W, T1 weighted; BOLD, blood-oxygen-level-dependent; DSI, diffusion spectrum imaging; QSM, quantitative susceptibility mapping; ASL, arterial spin labeling.

Supplementary Table 2 Perceived Stress Scale

	Never	Almost Never	Sometime	Fairly Often	Very Often
1. In the last month, how often have you been upset because of something that happened unexpectedly?	1	2	3	4	5
2. In the last month, how often have you felt that you were unable to control the important things in your life?	1	2	3	4	5
3. In the last month, how often have you felt nervous and “stressed”?	1	2	3	4	5
4. In the last month, how often have you dealt successfully with day to day problems and annoyances?	1	2	3	4	5
5. In the last month, how often have you felt that you were effectively coping with important changes that were occurring in your life?	1	2	3	4	5
6. In the last month, how often have you felt confident about your ability to handle your personal problems?	1	2	3	4	5
7. In the last month, how often have you felt that things were going your way?	1	2	3	4	5
8. In the last month, how often have you found that you could not cope with all the things that you had to do?	1	2	3	4	5
9. In the last month, how often have you been able to control irritations in your life?	1	2	3	4	5
10. In the last month, how often have you felt that you were on top of things?	1	2	3	4	5
11. In the last month, how often have you been angered because of things that were outside your control?	1	2	3	4	5
12. In the last month, how often have you found yourself thinking about things that you have to accomplish?	1	2	3	4	5
13. In the last month, how often have you been able to control the way you spend your time?	1	2	3	4	5
14. In the last month, how often have you felt difficulties were piling up so high that you could not overcome them?	1	2	3	4	5

Note.—Questionnaire completion guide: The questions in this scale ask you about your feelings and thoughts during the last month. In each case, you will be asked to indicate your response by placing an “X” over the circle representing HOW OFTEN you felt or thought a certain way. Although some of the questions are similar, there are differences between them and you should treat each one as a separate question. The best approach is to answer fairly quickly. That is, don’t try to count up the number of times you felt a particular way, but rather indicate the alternative that seems like a reasonable estimate.

Supplementary Table 3 State-Trait Anxiety Inventory (State score)

	NOT AT ALL	SOMEWHAT	MODERATE SO	VERY MUCH SO
1. I feel calm	1	2	3	4
2. I feel secure	1	2	3	4
3. I am tense	1	2	3	4
4. I feel strained	1	2	3	4
5. I feel at ease	1	2	3	4
6. I feel upset	1	2	3	4
7. I am presently worrying over possible misfortunes	1	2	3	4
8. I feel satisfied	1	2	3	4
9. I feel frightened	1	2	3	4
10. I feel comfortable	1	2	3	4
11. I feel self-confident	1	2	3	4
12. I feel nervous	1	2	3	4
13. I am jittery	1	2	3	4
14. I feel indecisive	1	2	3	4
15. I am relaxed	1	2	3	4
16. I feel content	1	2	3	4
17. I am worried	1	2	3	4
18. I feel confused	1	2	3	4
19. I feel steady	1	2	3	4
20. I feel pleasant	1	2	3	4

Note.—Questionnaire completion guide: A number of statements which people have used to describe themselves are given below. Read each statement and then circle the appropriate number to the right of the statement to indicate how you feel right now, that is, at this moment. There are no right or wrong answers. Do not spend too much time on any one statement but give the answer which seems to describe your present feelings best.

Supplementary Table 4 State-Trait Anxiety Inventory (Trait score)

	ALMOST NEVER	SOMETIMES	OFTEN	ALMOST ALWAYS
21. I feel pleasant	1	2	3	4
22. I feel nervous and restless	1	2	3	4
23. I feel satisfied with myself	1	2	3	4
24. I wish I could be as happy as others seem to be	1	2	3	4
25. I feel like a failure	1	2	3	4
26. I feel rested	1	2	3	4
27. I am "calm, cool, and collected"	1	2	3	4
28. I feel that difficulties are piling up so that I cannot overcome them	1	2	3	4
29. I worry too much over something that really doesn't matter	1	2	3	4
30. I am happy	1	2	3	4
31. I have disturbing thoughts	1	2	3	4
32. I lack self-confidence	1	2	3	4
33. I feel secure	1	2	3	4
34. I make decisions easily	1	2	3	4
35. I feel inadequate	1	2	3	4
36. I am content	1	2	3	4
37. Some unimportant thought runs through my mind and bothers me	1	2	3	4
38. I take disappointments so keenly that I can't put them out of my mind	1	2	3	4
39. I am a steady person	1	2	3	4
40. I get in a state of tension or turmoil as I think over my recent concerns and interests	1	2	3	4

Supplementary Table 5 Beck's Depression Inventory

Beck's Depression Inventory	
1.	
0	I do not feel sad.
1	I feel sad
2	I am sad all the time and I can't snap out of it.
3	I am so sad and unhappy that I can't stand it.
2.	
0	I am not particularly discouraged about the future.
1	I feel discouraged about the future.
2	I feel I have nothing to look forward to.
3	I feel the future is hopeless and that things cannot improve.
3.	
0	I do not feel like a failure.
1	I feel I have failed more than the average person.
2	As I look back on my life, all I can see is a lot of failures.
3	I feel I am a complete failure as a person.
4.	
0	I get as much satisfaction out of things as I used to.
1	I don't enjoy things the way I used to.
2	I don't get real satisfaction out of anything anymore.
3	I am dissatisfied or bored with everything.
5.	
0	I don't feel particularly guilty
1	I feel guilty a good part of the time.
2	I feel quite guilty most of the time.
3	I feel guilty all of the time.
6.	
0	I don't feel I am being punished.
1	I feel I may be punished.
2	I expect to be punished.
3	I feel I am being punished.
7.	
0	I don't feel disappointed in myself.
1	I am disappointed in myself.
2	I am disgusted with myself.
3	I hate myself.
8.	
0	I don't feel I am any worse than anybody else.
1	I am critical of myself for my weaknesses or mistakes.
2	I blame myself all the time for my faults.
3	I blame myself for everything bad that happens.

9.

0 I don't have any thoughts of killing myself.
1 I have thoughts of killing myself, but I would not carry them out.
2 I would like to kill myself.
3 I would kill myself if I had the chance.

10.

0 I don't cry any more than usual.
1 I cry more now than I used to.
2 I cry all the time now.
3 I used to be able to cry, but now I can't cry even though I want to.

11.

0 I am no more irritated by things than I ever was.
1 I am slightly more irritated now than usual.
2 I am quite annoyed or irritated a good deal of the time.
3 I feel irritated all the time.

12.

0 I have not lost interest in other people.
1 I am less interested in other people than I used to be.
2 I have lost most of my interest in other people.
3 I have lost all of my interest in other people.

13.

0 I make decisions about as well as I ever could.
1 I put off making decisions more than I used to.
2 I have greater difficulty in making decisions more than I used to.
3 I can't make decisions at all anymore.

14.

0 I don't feel that I look any worse than I used to.
1 I am worried that I am looking old or unattractive.
2 I feel there are permanent changes in my appearance that make me look unattractive
3 I believe that I look ugly.

15.

0 I can work about as well as before.
1 It takes an extra effort to get started at doing something.
2 I have to push myself very hard to do anything.
3 I can't do any work at all.

16.

0 I can sleep as well as usual.
1 I don't sleep as well as I used to.
2 I wake up 1-2 hours earlier than usual and find it hard to get back to sleep.
3 I wake up several hours earlier than I used to and cannot get back to sleep.

17.

0 I don't get more tired than usual.

1	I get tired more easily than I used to.
2	I get tired from doing almost anything.
3	I am too tired to do anything.
	18.
0	My appetite is no worse than usual.
1	My appetite is not as good as it used to be.
2	My appetite is much worse now.
3	I have no appetite at all anymore.
	19.
0	I haven't lost much weight, if any, lately.
1	I have lost more than five pounds.
2	I have lost more than ten pounds.
3	I have lost more than fifteen pounds.
	20.
0	I am no more worried about my health than usual.
1	I am worried about physical problems like aches, pains, upset stomach, or constipation.
2	I am very worried about physical problems and it's hard to think of much else.
3	I am so worried about my physical problems that I cannot think of anything else.
	21.
0	I have not noticed any recent change in my interest in sex.
1	I am less interested in sex than I used to be.
2	I have almost no interest in sex.
3	I have lost interest in sex completely.

Supplementary Table 6 Relative abundance of the 24 CD-enriched bacteria genera

**Taxa**	**Relative abundance**	**LDA score**	***P* value**
Bacteria.Proteobacteria.Gammaproteobacteria.Enterobacteriales.Enterobacteriaceae.Escherichia_Shigella	5.30	4.88	<0.001
Bacteria.Firmicutes.Clostridia.Clostridiales.Lachnospiraceae._Ruminococcus__gnavus_group	4.72	4.35	<0.001
Bacteria.Firmicutes.Clostridia.Clostridiales.Lachnospiraceae.Lachnoclostridium	4.54	3.84	0.04
Bacteria.Firmicutes.Bacilli.Lactobacillales.Enterococcaceae.Enterococcus	4.43	4.15	<0.001
Bacteria.Firmicutes.Bacilli.Lactobacillales.Lactobacillaceae.Lactobacillus	4.38	4.01	<0.001
Bacteria.Fusobacteria.Fusobacteriia.Fusobacteriales.Fusobacteriaceae.Fusobacterium	4.36	3.97	0.02
Bacteria.Firmicutes.Negativicutes.Selenomonadales.Veillonellaceae.Veillonella	4.29	3.96	<0.001
Bacteria.Firmicutes.Erysipelotrichia.Erysipelotrichales.Erysipelotrichaceae.Erysipelatoclostridium	4.27	3.94	<0.001
Bacteria.Actinobacteria.Coriobacteriia.Coriobacteriales.Coriobacteriaceae.Collinsella	4.18	3.40	<0.01
Bacteria.Actinobacteria.Actinobacteria.Actinomycetales.Actinomycetaceae.Actinomyces	3.75	3.47	<0.001
Bacteria.Firmicutes.Clostridia.Clostridiales.Peptostreptococcaceae.Peptostreptococcus	3.69	3.33	<0.001
Bacteria.Firmicutes.Erysipelotrichia.Erysipelotrichales.Erysipelotrichaceae._Clostridium__innocuum_group	3.65	3.32	<0.001
Bacteria.Firmicutes.Clostridia.Clostridiales.Lachnospiraceae._Ruminococcus__torques_group	3.61	2.78	0.01
Bacteria.Firmicutes.Clostridia.Clostridiales.Lachnospiraceae.Tyzzerella_4	3.60	3.03	0.03
Bacteria.Actinobacteria.Coriobacteriia.Coriobacteriales.Eggerthellaceae.Eggerthella	3.48	3.08	0.01
Bacteria.Firmicutes.Bacilli.Lactobacillales.Carnobacteriaceae.Granulicatella	3.34	3.03	<0.01
Bacteria.Firmicutes.Bacilli.Bacillales.Family_XI.Gemella	3.27	2.98	<0.001
Bacteria.Patescibacteria.Saccharimonadia.Saccharimonadales	3.18	2.88	<0.001
Bacteria.Patescibacteria.Saccharimonadia.Saccharimonadales.Saccharimonadaceae.Saccharimonadaceae	3.14	2.84	<0.001
Bacteria.Firmicutes.Erysipelotrichia.Erysipelotrichales.Erysipelotrichaceae.Faecalitalea	3.09	2.72	<0.01
Bacteria.Firmicutes.Erysipelotrichia.Erysipelotrichales.Erysipelotrichaceae.Solobacterium	2.95	2.44	<0.001
Bacteria.Firmicutes.Clostridia.Clostridiales.Lachnospiraceae.Lachnospiraceae_UCG_010	2.90	2.29	<0.001
Bacteria.Actinobacteria.Coriobacteriia.Coriobacteriales.Atopobiaceae.Atopobium	2.70	2.38	<0.001
Bacteria.Firmicutes.Clostridia.Clostridiales.Lachnospiraceae.Oribacterium	2.49	2.22	0.01

Note.—LDA, Linear Discriminant Analysis.

Supplementary Table 7 Pearson correlation coefficients between brain radiomics features and blood metabolites

	Brain-Stem fMRI ALFF p90	Ctx-Lh-Caudal-Anterior-Cingulate DKI KFA std	Ctx-Lh-Cuneus fMRI ReHo std	Left-Hippocampus R2Star p10	Ctx-Lh-Middle Temporal CBF p90	Ctx-Rh-Postcentral fMRI ReHo p90	Left-Putamen fMRI ReHo p10	Ctx-Rh-SuperiorFrontal DKI GA std	Ctx-Lh-Superior Temporal vol	Left-Thalamus-Proper CBF std	Ctx-Lh-Middle Temporal vol	Ctx-Rh-Precentral fMRI ALFF p10	Ctx-Rh-Precentral fMRI ReHo mean
10_13_Nonadecadienoic_acid	-0.30	0.08	-0.02	-0.42	0.10	-0.03	-0.22	0.02	0.04	-0.20	0.20	0.26	0.23
12_Tridecenoic_acid	-0.29	0.10	0.13	-0.14	-0.03	-0.01	0.03	0.26	0.13	-0.07	0.10	0.18	0.19
2_Furoic_acid	-0.09	0.11	-0.05	0.02	-0.25	0.10	0.03	-0.15	0.16	-0.28	-0.26	0.04	-0.03
2_Methylhexanoic_acid	0.17	0.36	0.12	-0.14	-0.24	0.16	-0.05	-0.15	-0.03	-0.38	-0.22	0.24	0.08
2_Phenylpropionate	-0.23	-0.07	-0.03	-0.33	-0.24	0.14	0.06	-0.04	-0.25	-0.28	-0.30	0.10	0.12
3_Methyladipic_acid	-0.26	-0.06	-0.01	-0.07	0.38	-0.01	-0.11	0.10	0.02	0.05	-0.12	0.11	0.14
4_Aminohippuric_acid	-0.13	0.02	-0.10	-0.08	-0.01	0.02	0.00	-0.16	0.06	-0.06	0.00	0.11	0.27
4_Methylhexanoic_acid	-0.04	0.15	0.21	-0.12	-0.21	0.08	-0.14	-0.15	-0.03	-0.30	-0.26	0.19	0.09
Aminocaproic_acid	-0.10	-0.08	-0.07	-0.11	0.11	-0.18	-0.21	-0.11	0.01	0.20	0.12	0.01	0.12
AMP	-0.35	0.11	-0.01	0.02	-0.04	0.12	0.08	-0.03	-0.01	0.00	-0.19	0.05	-0.05
Arginine	0.03	0.00	-0.30	-0.10	0.13	-0.07	-0.17	-0.37	-0.05	-0.13	-0.12	0.02	0.09
Aspartic_acid	0.00	-0.06	0.04	-0.04	0.08	-0.05	-0.29	-0.09	-0.35	-0.01	-0.10	0.00	0.04
Azelaic_acid	-0.06	-0.10	0.00	0.10	0.11	0.00	-0.12	0.19	-0.35	0.11	-0.15	0.02	-0.04
Butyrylcarnitine	0.07	0.00	0.30	0.07	0.10	0.15	-0.17	-0.11	0.04	0.05	0.13	0.20	0.14
CA	-0.09	-0.09	-0.27	0.14	0.18	-0.11	0.10	-0.12	-0.02	0.11	-0.21	-0.06	-0.12
CDCA	-0.05	-0.23	-0.30	0.20	0.14	-0.18	-0.07	-0.01	-0.14	0.17	-0.14	-0.11	-0.12
CE_16_1_	-0.09	0.39	0.06	-0.13	-0.17	0.02	0.25	0.06	0.20	-0.02	0.22	0.08	-0.10
Cer_d18_0_22_0_	-0.02	-0.16	0.01	0.04	-0.02	0.10	-0.01	-0.07	0.06	-0.16	0.37	0.12	0.09
Cer_d18_0_24_0_	-0.02	-0.05	-0.01	0.00	-0.08	0.00	0.18	0.07	0.16	-0.27	-0.02	0.05	0.03
Cer_d18_0_24_1_	0.15	-0.15	-0.03	0.00	0.14	0.00	-0.12	-0.14	0.03	0.02	0.39	-0.08	0.04
Cer_d18_1_22_0_	0.10	-0.01	0.22	0.08	-0.09	-0.02	0.02	-0.11	0.26	-0.21	-0.02	-0.04	0.01
Cer_d18_2_22_0_	0.19	0.03	0.26	0.08	-0.16	0.14	0.17	0.03	0.30	-0.15	-0.10	0.01	0.04

Cinnamic_acid	-0.04	-0.27	-0.02	-0.21	0.14	0.08	-0.05	0.23	-0.09	0.07	0.07	0.07	0.08
Citraconic_acid	0.07	0.07	0.15	-0.33	0.06	-0.18	-0.26	0.21	-0.09	-0.02	-0.06	0.10	0.06
Citramalic_acid	-0.03	-0.11	0.24	0.15	-0.09	0.19	0.27	0.30	0.08	-0.02	-0.19	0.03	-0.15
Citrulline	0.07	-0.31	-0.35	0.16	0.09	-0.13	0.11	0.08	0.08	-0.04	-0.17	-0.05	0.01
DAG_32_0_	0.21	-0.32	0.01	-0.06	0.07	0.02	-0.20	-0.19	-0.06	0.07	-0.04	0.00	0.09
DHA	-0.19	0.33	0.13	-0.23	0.02	0.07	-0.14	0.07	-0.01	-0.10	-0.06	0.36	0.31
Dimethylglycine	-0.02	0.00	0.06	-0.35	0.28	0.09	-0.21	-0.38	-0.25	0.10	-0.18	0.04	0.21
DPA	-0.15	0.04	0.17	-0.13	-0.02	0.15	-0.16	-0.08	0.12	-0.02	0.13	0.31	0.34
ePE_34_1_	-0.03	0.01	-0.02	-0.24	0.06	-0.08	-0.01	0.10	-0.06	-0.28	-0.05	-0.13	0.03
ePE_34_2_	-0.02	-0.06	-0.17	-0.29	0.06	-0.11	-0.06	0.25	-0.14	-0.26	-0.17	0.03	0.11
ePE_36_3_	-0.04	-0.13	-0.23	-0.27	0.05	-0.16	-0.12	0.24	-0.11	-0.27	-0.10	-0.07	0.07
ePE_38_1_	-0.10	0.13	0.07	-0.01	-0.04	-0.08	0.17	-0.02	0.26	-0.21	-0.12	-0.04	-0.15
gamma_Linolenic_acid	-0.07	0.14	0.22	-0.16	-0.03	0.13	-0.14	-0.03	0.15	-0.07	0.17	0.38	0.34
GCDCA_3S	0.01	0.29	-0.08	-0.22	0.10	0.24	-0.23	-0.21	-0.07	-0.16	0.18	0.27	0.22
Glutamine	0.04	-0.24	-0.36	0.15	0.05	-0.02	0.08	0.15	-0.02	0.05	-0.06	0.10	0.13
Glutarylcarnitine	0.03	-0.37	0.06	-0.09	0.13	-0.01	-0.07	-0.12	0.06	0.17	-0.05	-0.08	0.35
Glyceraldehyde	0.02	-0.08	-0.05	0.12	-0.12	-0.12	-0.13	-0.07	0.27	0.02	-0.02	-0.06	-0.01
Guanidoacetic_acid	-0.04	0.00	0.08	0.15	-0.15	0.16	0.46	0.22	0.14	-0.23	-0.16	0.17	0.01
Heptanoic_acid	-0.09	-0.12	-0.02	0.09	0.21	-0.03	-0.20	0.16	-0.32	0.10	-0.08	0.07	0.00
Homocitrulline	-0.01	0.00	-0.04	0.09	0.07	-0.15	-0.06	-0.14	0.27	0.04	-0.04	-0.18	-0.04
Homoserine	-0.32	-0.08	0.08	0.19	0.24	0.07	0.30	0.17	-0.14	0.14	-0.17	0.16	0.04
Homovanillic_acid	0.06	0.24	0.08	-0.20	-0.02	0.19	0.10	0.14	-0.15	-0.05	-0.09	0.15	0.13
Hydrocinnamic_acid	-0.20	-0.08	0.01	-0.32	-0.24	0.08	0.07	-0.03	-0.21	-0.28	-0.26	0.07	0.11
Indole_3_propionic_acid	0.10	-0.17	-0.11	0.06	0.01	-0.24	0.06	-0.06	0.00	0.04	0.22	-0.10	0.05
Indolelactic_acid	0.02	0.08	0.21	-0.01	-0.01	0.32	0.05	0.22	-0.12	0.05	-0.08	0.21	0.09
Isocaproic_acid	-0.10	0.02	-0.08	0.06	0.03	0.13	0.13	0.16	0.07	-0.01	0.06	0.22	0.34
Isocitric_acid	-0.07	-0.13	0.12	0.06	-0.02	0.10	0.12	0.10	0.26	-0.05	0.03	0.18	0.08
Isoleucine	-0.04	-0.17	-0.21	-0.01	0.02	0.13	-0.09	-0.01	-0.29	0.02	-0.06	0.20	0.26
Linoleic_acid	-0.09	0.00	0.05	0.08	0.06	0.10	0.03	0.02	0.20	0.05	0.29	0.07	0.08

LPC_16_1_	0.27	-0.08	0.15	0.10	-0.25	0.16	0.09	0.15	0.09	-0.01	-0.12	0.08	0.08
LPC_18_2_	0.10	-0.24	-0.05	0.08	-0.09	0.21	0.06	0.21	0.04	0.00	-0.29	0.05	0.14
LPC_20_0_	0.07	-0.27	-0.05	0.18	-0.14	0.03	0.07	0.19	0.02	-0.10	-0.13	-0.07	-0.02
LPC_20_1_	0.23	-0.25	-0.06	0.05	-0.03	0.00	-0.15	0.05	-0.05	0.00	-0.16	-0.17	0.02
LPC_20_5_	0.02	-0.21	0.15	0.24	0.03	0.03	0.02	0.23	-0.02	0.06	0.00	0.01	0.00
LPC_22_6_	-0.07	-0.18	0.04	0.20	-0.03	0.07	0.18	0.23	0.08	0.18	-0.16	0.00	0.08
LPC_28_1_	0.20	0.14	0.31	-0.02	-0.06	0.22	0.19	0.10	-0.03	-0.05	-0.10	0.09	0.05
LPE_18_1_	0.31	-0.18	0.04	0.00	-0.04	0.11	-0.08	0.15	0.09	0.06	-0.21	-0.04	0.07
LPE_18_2_	0.30	-0.20	-0.11	0.01	-0.08	0.08	-0.13	0.10	0.01	-0.07	-0.20	-0.10	0.04
LPE_18_3_	0.35	-0.15	0.18	0.00	-0.05	0.09	-0.06	0.03	0.05	-0.04	-0.18	-0.12	0.06
LPE_22_6_	0.04	-0.09	0.05	0.09	0.07	0.03	0.07	0.17	0.03	0.29	-0.20	-0.01	0.15
Mandelic_acid	0.07	0.00	0.14	0.11	-0.06	0.32	-0.09	-0.02	0.12	0.17	0.05	0.11	0.01
Methionine	-0.15	-0.15	-0.07	0.26	-0.18	0.00	0.25	0.26	-0.13	0.00	0.06	-0.05	-0.05
Methylglutaric_acid	0.04	0.00	-0.08	-0.04	-0.17	0.08	0.06	0.06	0.17	-0.28	-0.01	-0.03	-0.04
Myristoleic_acid	-0.14	0.26	0.08	-0.30	-0.08	0.13	-0.06	0.02	0.26	-0.15	0.14	0.31	0.23
N_Methylnicotinamide	-0.12	-0.02	-0.17	0.03	-0.09	-0.05	0.04	-0.26	0.13	-0.08	-0.18	-0.09	-0.03
Oleic_acid	-0.16	0.12	0.07	-0.31	0.06	0.01	-0.10	0.00	0.22	-0.13	0.30	0.17	0.11
p_Hydroxyphenylacetic_acid	-0.04	-0.29	-0.02	0.17	0.17	-0.19	-0.08	0.08	-0.04	0.27	-0.05	-0.22	-0.03
Palmitoleic_acid	-0.04	0.25	0.02	-0.19	-0.05	0.14	-0.13	-0.12	0.17	-0.11	0.14	0.27	0.28
PC_26_0_	0.28	-0.10	0.01	-0.18	0.14	0.19	-0.09	-0.03	0.01	0.10	0.12	-0.15	0.04
PC_30_0_	0.30	0.07	0.21	-0.02	-0.06	0.20	0.16	0.10	0.12	0.06	0.10	-0.03	-0.04
PC_32_1_	0.23	0.29	0.22	-0.03	-0.21	0.07	0.24	0.06	0.26	0.00	0.18	-0.01	-0.08
PC_32_2_	0.29	-0.02	0.14	0.04	-0.18	0.15	0.15	0.16	0.09	-0.14	0.05	-0.11	-0.04
PC_32_3_	0.33	-0.07	0.16	0.02	-0.08	0.15	0.14	0.17	0.07	-0.01	0.08	-0.14	-0.03
PC_34_3_	0.22	0.09	0.05	0.05	-0.32	-0.04	0.09	0.10	0.18	-0.25	-0.02	-0.12	-0.03
PC_34_4_	0.16	0.00	0.28	0.09	-0.16	0.15	0.26	0.19	0.06	-0.07	0.06	-0.04	-0.01
PC_36_0_	0.08	0.14	0.29	-0.04	-0.18	0.07	0.06	-0.03	0.21	-0.10	0.05	0.05	-0.05
PC_38_0_	-0.12	-0.18	-0.04	0.14	0.00	-0.02	0.33	0.29	0.15	-0.08	0.37	0.12	-0.04
PC_40_8_	-0.12	0.01	0.11	0.21	-0.11	0.04	0.28	0.18	0.14	-0.03	-0.06	-0.04	-0.02

PC_42_10_	-0.18	0.06	0.16	0.23	-0.08	0.04	0.35	0.25	0.16	-0.01	-0.02	0.00	-0.06
PC_42_9_	-0.03	0.10	0.28	0.21	-0.16	0.07	0.28	0.20	0.16	-0.02	0.00	0.03	-0.05
PC_44_11_	0.03	0.01	0.09	-0.09	0.05	-0.20	-0.19	-0.12	0.10	0.00	0.26	-0.03	0.12
PC_44_12_	0.01	0.02	0.09	-0.10	0.07	-0.18	-0.20	-0.12	0.10	0.03	0.25	-0.01	0.15
PC_O_30_2_	0.02	0.14	0.20	0.04	-0.18	0.33	0.25	0.08	0.08	-0.09	0.00	0.29	0.21
PC_O_34_3_	-0.28	0.25	0.20	-0.06	-0.11	0.12	0.32	0.25	0.02	-0.24	-0.02	0.09	-0.04
PC_O_36_4_	-0.19	0.26	0.27	-0.07	-0.02	0.02	0.16	0.02	-0.04	0.04	-0.04	0.11	0.05
PC_O_38_6_	-0.28	0.26	0.16	0.03	0.05	-0.01	0.26	0.07	0.01	0.04	0.02	0.02	-0.06
PC_O_42_4_	-0.11	0.02	0.06	0.07	0.09	-0.24	0.03	0.04	0.07	0.07	0.25	-0.02	0.00
PC_O_42_5_	0.04	0.06	0.03	-0.10	0.08	-0.21	-0.15	-0.15	0.04	0.10	0.26	-0.09	0.09
PE_32_1_	0.32	0.35	0.10	-0.19	-0.08	-0.04	-0.08	-0.14	0.19	-0.09	0.18	-0.14	-0.01
PE_34_2_	0.39	0.11	0.00	-0.22	-0.02	-0.01	-0.21	-0.18	0.09	-0.13	0.07	-0.19	0.09
PE_34_3_	0.43	0.01	0.07	-0.21	0.03	0.00	-0.17	-0.12	0.08	-0.05	0.08	-0.24	0.05
PE_36_2_	0.41	0.04	0.02	-0.16	-0.05	-0.01	-0.24	-0.20	0.09	-0.12	0.11	-0.17	0.09
PE_38_5_	0.29	0.13	0.17	-0.14	-0.01	-0.01	-0.21	-0.19	0.06	0.01	0.04	-0.11	0.16
PE_40_3_	0.17	0.00	0.13	0.08	-0.05	-0.07	0.09	-0.02	0.02	0.09	0.28	-0.07	0.03
PE_40_5_	0.28	0.10	0.19	-0.07	-0.11	0.03	-0.15	-0.21	0.05	0.00	0.14	0.00	0.19
Pentadecanoic_acid	-0.23	0.62	0.10	-0.09	-0.03	-0.18	0.11	0.02	0.23	-0.24	-0.08	0.07	-0.14
Phenylacetylglutamine	-0.09	-0.07	-0.25	0.06	-0.07	-0.06	0.13	-0.22	0.08	-0.10	-0.24	-0.10	-0.06
Phenylalanine	-0.03	-0.19	0.10	0.03	0.07	0.06	-0.27	-0.08	-0.39	0.06	0.02	0.11	0.23
Phenyllactic_acid	-0.02	-0.05	0.23	0.13	-0.11	0.19	0.09	0.25	-0.05	0.18	-0.04	0.02	-0.10
Phenylpyruvic_acid	0.24	0.01	0.21	-0.07	0.12	0.21	-0.29	-0.14	-0.20	0.12	0.01	0.28	0.28
PI_34_1_	0.29	0.05	0.13	-0.16	-0.02	0.28	0.07	0.02	0.07	0.16	0.02	0.10	0.05
PI_34_2_	0.44	0.01	-0.02	-0.10	-0.14	0.15	-0.05	-0.18	0.03	-0.09	0.10	0.04	0.07
PI_36_2_	0.38	-0.11	-0.08	0.00	-0.24	0.03	-0.14	-0.16	0.01	-0.17	-0.01	-0.06	-0.04
PI_36_3_	0.38	0.11	0.09	-0.01	-0.20	0.21	0.00	-0.07	0.00	-0.05	-0.11	0.10	0.10
PI_36_4_	0.31	0.24	0.23	-0.05	-0.17	0.22	0.06	-0.12	0.15	-0.02	0.07	0.22	0.14
PI_38_5_	0.41	0.04	0.21	-0.02	-0.07	0.20	-0.14	-0.10	0.03	0.03	-0.07	0.19	0.22
PI_38_6_	0.37	0.19	0.12	-0.06	0.04	0.13	-0.10	-0.23	0.11	0.28	0.17	0.21	0.18

PI_40_6_	0.07	0.23	0.07	0.13	-0.22	0.14	0.20	-0.13	0.25	-0.09	0.14	0.19	-0.01
Pimelic_acid	-0.05	-0.11	-0.04	0.07	0.10	-0.03	-0.15	0.17	-0.33	0.07	-0.16	0.00	-0.05
PS_32_1_	0.20	0.13	0.16	0.06	-0.18	0.07	0.26	0.16	0.23	0.06	0.25	0.00	-0.09
PS_34_4_	0.20	0.05	0.15	0.17	-0.32	0.02	0.20	0.12	0.15	-0.22	0.04	-0.10	-0.08
PS_36_6_	0.01	-0.09	0.13	0.24	-0.02	-0.09	0.08	0.10	0.06	-0.10	0.31	-0.05	-0.13
PS_40_8_	-0.16	-0.05	0.18	0.27	-0.15	0.22	0.47	0.23	-0.07	0.02	0.25	0.24	-0.03
Pyroglutamic_acid	-0.03	-0.23	-0.21	0.19	0.05	-0.02	0.02	-0.06	-0.28	-0.04	-0.21	-0.04	-0.06
Pyruvic_acid	0.09	0.01	0.04	0.02	-0.43	0.20	0.15	-0.05	0.04	-0.33	-0.22	0.05	-0.08
Ribulose	-0.20	0.03	-0.05	-0.08	0.12	-0.01	0.18	-0.02	-0.18	-0.04	-0.33	-0.13	-0.04
Ricinoleic_acid	-0.19	0.24	-0.02	-0.20	0.00	0.01	-0.04	0.13	0.28	-0.23	0.18	0.17	0.10
Sebacic_acid	-0.06	-0.11	0.01	0.10	0.13	0.00	-0.13	0.18	-0.36	0.13	-0.15	0.03	-0.03
SM_d16_1_22_2_	-0.02	0.15	-0.04	0.06	-0.28	-0.02	-0.22	-0.14	-0.01	-0.21	-0.24	-0.01	-0.08
SM_d18_0_12_0_	0.33	-0.04	0.10	-0.11	0.02	0.16	0.05	0.09	0.05	-0.03	0.03	-0.18	-0.05
SM_d18_1_19_0_	-0.10	0.01	-0.02	-0.29	-0.02	-0.09	-0.06	0.22	-0.10	-0.27	-0.19	0.02	0.04
Suberic_acid	-0.06	-0.09	0.01	0.11	0.08	0.01	-0.11	0.19	-0.34	0.06	-0.15	0.03	-0.05
Succinic_acid	-0.18	-0.19	-0.07	-0.13	0.11	-0.04	-0.06	0.20	0.05	0.05	0.02	-0.08	0.07
TG_45_0_	-0.22	0.18	-0.13	-0.19	-0.07	-0.14	-0.12	-0.14	0.03	-0.28	-0.09	-0.12	0.02
TG_49_3_	-0.09	-0.09	-0.10	-0.16	0.13	-0.17	-0.29	-0.04	0.00	-0.10	0.09	0.06	0.21
TG_52_0_	-0.10	-0.06	-0.11	-0.27	0.28	-0.08	-0.15	0.08	-0.11	0.15	0.01	-0.05	0.05
TG_52_6_	-0.02	-0.21	-0.01	-0.26	0.23	-0.14	-0.26	0.07	-0.04	0.06	0.08	0.06	0.21
TG_54_7_	-0.13	-0.08	-0.15	-0.22	0.32	-0.17	-0.19	0.06	-0.14	0.09	0.07	-0.04	0.10
TG_56_5_	-0.02	-0.01	0.11	0.08	0.11	-0.10	-0.06	-0.28	-0.07	0.08	0.21	0.04	0.14
TG_56_7_	0.17	-0.11	0.25	0.04	-0.04	0.05	-0.15	-0.11	-0.01	0.05	0.05	0.11	0.31
UDCA	-0.07	0.00	-0.30	0.09	-0.01	-0.04	0.04	-0.06	-0.01	0.09	-0.36	-0.05	-0.13
Undecanoic_acid	-0.08	-0.17	0.01	-0.03	0.17	-0.04	-0.21	0.18	-0.26	0.13	-0.13	0.02	0.01
Undecylenic_acid	-0.21	0.11	0.07	-0.35	0.09	-0.05	-0.15	0.00	-0.05	0.05	0.11	0.09	0.18
Valeric_acid	0.00	-0.17	-0.07	0.02	0.21	0.09	-0.18	0.00	-0.32	0.16	-0.15	0.16	0.07
Xylose	-0.20	0.17	-0.22	-0.14	0.23	0.01	0.01	-0.05	-0.11	0.05	-0.25	-0.06	-0.11

Note.—AMP, Adenosine monophosphate; CA, cholic acid; CDCA, chenodeoxycholic acid; CE, Cholesteryl ester; CER, Ceramide;

DAG, Diacylglycero; DHA, Docosahexaenoic acid; DPA, Docosapentaenoic acid; EPA, Eicosapentaenoic acid; GCDCA_s, glycochenodeoxycholic Acid Sulfate; LPC, lysoPhosphatidylcholine; LPE, lysoPhosphatidylethanolamine; PC, Phosphatidylcholine; PE, Phosphatidylethanolamine; PI, Phosphatidylinositol; PS, Phosphatidylserine; UDCA, ursodeoxycholic acid; SM, Sphingomyelin; TG, Triacylglycerol.

Supplementary Table 8 Pearson correlation coefficients between CD-enriched bacteria genera and blood metabolites

	Actinomyces	Collinsella	Enterococcus	Faecalitalea	Fusobacterium	LachnospiraceaeUCG010	Oribacterium	Saccharimonadales	Veillonella
10_13_Nonadecadienoic_acid	0.19	0.11	-0.02	0.44	0.05	0.12	-0.11	-0.25	0.03
10Z_Nonadecenoic_acid	0.18	-0.08	0.14	0.46	0.05	-0.10	-0.20	-0.34	-0.03
2_2_Dimethyladipic_acid	0.36	0.25	-0.04	-0.03	-0.21	-0.07	0.01	0.11	0.00
2_Hydroxyglutaric_acid	-0.19	-0.20	0.22	0.06	-0.15	-0.19	0.00	-0.06	-0.01
2_Methylpentanoic_acid	0.22	-0.02	0.27	0.31	-0.07	-0.15	-0.22	-0.32	-0.12
4_Hydroxyproline	-0.18	0.04	-0.01	0.02	0.34	0.05	-0.19	0.13	0.06
5Z_Dodecenoic_acid	0.16	0.23	-0.04	0.40	-0.11	-0.02	-0.14	-0.25	-0.11
9E_tetradecenoic_acid	0.11	0.30	-0.02	0.43	-0.07	0.01	-0.11	-0.22	-0.08
Acetic_acid	0.10	-0.07	-0.04	0.04	-0.03	-0.14	-0.05	-0.26	0.02
Acetylcarnitine	0.09	-0.05	0.11	0.18	-0.08	-0.10	-0.07	-0.27	0.00
Alanine	-0.18	-0.01	0.15	0.01	-0.19	0.14	0.00	0.25	-0.25
alpha_Ketoisovaleric_acid	0.09	0.08	0.11	0.18	-0.10	-0.03	-0.26	-0.20	-0.07
alpha_Linolenic_acid	0.33	0.04	0.04	0.21	-0.09	-0.09	-0.17	-0.19	0.01
Aminoadipic_acid	0.15	0.03	0.01	0.14	-0.02	0.02	-0.21	-0.26	-0.17
Aspartic_acid	-0.01	-0.08	0.31	0.24	0.05	0.21	0.09	0.07	-0.04
Benzoic_acid	0.46	0.07	-0.07	-0.07	-0.09	0.16	-0.10	0.14	-0.14
bUDCA	-0.23	0.01	-0.09	-0.07	0.27	0.04	-0.18	0.11	-0.21
Butyric_acid	0.48	0.02	-0.10	-0.09	-0.11	-0.13	-0.10	-0.02	-0.12
CA	-0.11	-0.02	0.01	0.00	-0.03	-0.07	-0.06	-0.08	0.37
Capric_acid	0.06	0.06	0.18	0.21	-0.18	-0.15	-0.13	-0.32	-0.07
Caproic_acid	0.16	0.10	0.03	0.29	-0.29	-0.20	-0.07	-0.27	-0.12
Caprylic_acid	0.11	0.15	0.05	0.31	-0.25	-0.18	-0.12	-0.29	-0.05
CDCA	-0.10	-0.08	0.34	0.04	-0.10	-0.10	-0.10	-0.10	0.24
Cer_d16_1_23_0_	-0.19	-0.07	-0.12	-0.14	0.30	0.01	-0.16	0.12	-0.13
Cer_d18_0_24_1_	-0.03	-0.27	0.25	-0.01	-0.03	-0.10	0.04	-0.20	0.10
Cer_d18_2_24_2_	-0.14	-0.20	0.03	-0.08	0.27	-0.06	-0.06	0.10	-0.05

DAG_34_2_	0.17	-0.07	0.15	-0.22	-0.11	0.16	0.27	0.11	-0.03
Dodecanoic_acid	0.15	-0.16	0.12	-0.01	-0.19	-0.16	-0.19	-0.28	-0.18
Dodecanoylcarnitine	0.02	0.14	0.04	0.26	-0.02	-0.01	-0.04	-0.26	-0.06
EPA	-0.01	0.02	-0.13	0.05	0.27	0.01	-0.24	-0.10	-0.02
ePE_34_1_	-0.17	-0.29	0.14	0.06	0.11	-0.24	-0.19	0.02	0.08
ePE_36_1_	-0.27	-0.23	-0.12	0.25	0.09	-0.13	-0.11	0.01	0.14
ePE_36_5_	-0.19	-0.12	-0.21	-0.06	0.46	-0.09	-0.15	0.15	0.19
ePE_40_6_	-0.02	0.00	0.01	-0.14	0.23	0.16	-0.05	-0.09	0.01
Erythronic_acid	-0.09	-0.24	0.26	0.34	-0.04	-0.15	-0.14	-0.18	-0.17
Ethylmethylacetic_acid	0.28	0.04	0.15	-0.11	-0.30	-0.19	-0.03	0.02	0.11
Fructose	0.20	-0.11	0.01	-0.03	-0.04	-0.05	0.85	0.62	0.32
Fumaric_acid	-0.11	-0.06	0.27	0.15	-0.16	0.15	-0.31	-0.46	-0.27
GCDCA_3S	0.68	0.00	-0.02	0.10	0.10	-0.12	0.34	0.03	0.18
Gluconolactone	0.48	0.02	-0.02	0.08	0.02	0.17	0.17	0.17	0.03
Glucose	0.08	-0.04	0.17	0.13	-0.09	-0.03	0.32	0.33	0.10
Glutarylcarnitine	0.03	-0.27	0.35	0.02	-0.04	0.09	-0.05	-0.11	-0.01
Homoserine	-0.15	0.13	-0.12	-0.01	-0.16	-0.12	-0.14	0.26	-0.16
Ketoleucine	-0.04	0.12	0.09	0.19	-0.10	0.04	-0.26	-0.19	-0.10
Leucine	-0.13	-0.04	0.14	0.06	-0.05	0.08	-0.34	-0.22	-0.23
LPC_14_0_	-0.02	-0.04	0.36	-0.12	-0.19	-0.02	-0.08	0.09	-0.22
LPC_18_2_	-0.25	-0.24	0.29	0.06	-0.06	-0.23	-0.19	0.08	-0.18
LPC_18_3_	-0.07	-0.09	0.41	-0.09	-0.11	-0.07	-0.09	0.09	-0.11
LPC_20_3_	-0.16	-0.09	0.26	-0.11	-0.20	-0.08	-0.08	0.10	-0.14
LPC_20_4_	-0.26	-0.23	0.11	-0.08	-0.04	-0.12	-0.11	0.08	-0.08
LPC_22_5_	-0.23	-0.20	0.12	-0.03	-0.10	-0.19	-0.15	0.04	-0.22
LPC_22_6_	-0.28	-0.17	0.02	0.02	-0.03	-0.21	-0.21	0.03	-0.25
LPC_24_0_	0.01	-0.03	-0.27	0.15	-0.12	-0.24	0.10	-0.05	-0.01
LPC_26_0_	0.20	-0.08	-0.08	-0.08	-0.16	-0.17	0.27	0.00	0.24
LPC_28_0_	0.01	-0.21	0.31	0.16	-0.03	-0.26	-0.22	-0.22	-0.30

LPE_16_0_	0.03	-0.17	0.40	-0.08	-0.15	-0.15	-0.13	-0.01	-0.18
LPE_16_1_	0.06	0.03	0.32	-0.02	-0.22	-0.12	-0.14	0.08	-0.08
LPE_18_1_	0.01	-0.17	0.50	0.00	-0.21	-0.19	-0.06	0.06	-0.14
LPE_20_3_	-0.06	-0.07	0.41	-0.14	-0.21	-0.04	-0.08	0.07	-0.10
LPE_20_5_	0.04	-0.17	0.39	-0.19	-0.10	-0.20	-0.13	0.05	-0.02
Maleic_acid	0.22	-0.13	0.06	0.06	-0.21	0.01	-0.09	-0.35	-0.07
Malic_acid	-0.14	-0.08	0.24	0.18	-0.23	0.04	-0.14	-0.25	-0.06
Melatonin	-0.08	0.24	0.00	-0.03	-0.04	0.52	0.04	0.16	0.02
Myristic_acid	0.47	-0.09	-0.02	0.06	-0.19	-0.17	-0.17	-0.23	-0.06
Myristoleic_acid	0.25	-0.01	-0.10	0.29	-0.10	-0.06	-0.21	-0.28	-0.16
N_Acetylneuraminic_acid	0.02	-0.03	0.22	0.51	0.02	0.10	-0.14	-0.09	-0.09
Oleylcarnitine	-0.02	-0.14	0.06	0.29	-0.05	-0.02	-0.21	-0.37	-0.04
Oxoadipic_acid	-0.13	-0.25	0.22	0.15	0.08	-0.05	-0.14	-0.29	-0.03
p_Hydroxyphenylacetic_acid	-0.18	-0.13	0.51	-0.14	-0.10	-0.08	-0.13	-0.04	0.15
Palmitoleic_acid	0.29	-0.08	0.05	0.14	-0.07	-0.09	-0.19	-0.27	0.00
PC_24_0_	-0.05	-0.12	-0.26	0.03	0.00	-0.12	0.26	0.04	0.13
PC_26_0_	0.08	-0.14	0.61	-0.08	-0.14	-0.11	-0.04	-0.11	-0.11
PC_30_0_	0.13	-0.05	0.41	-0.15	-0.25	-0.13	-0.06	0.02	-0.21
PC_32_2_	0.03	-0.11	0.43	-0.09	-0.18	-0.04	-0.12	-0.01	-0.19
PC_32_3_	0.04	-0.06	0.49	-0.12	-0.14	-0.06	-0.09	0.04	-0.12
PC_34_0_	-0.15	-0.25	0.01	0.10	0.02	-0.08	0.04	0.05	-0.04
PC_34_4_	-0.06	-0.07	0.25	-0.14	-0.14	0.00	-0.10	0.08	-0.15
PC_40_2_	-0.04	-0.18	0.02	0.13	0.09	-0.01	-0.09	-0.26	0.01
PC_42_11_	0.03	-0.01	0.01	-0.07	-0.08	-0.17	0.10	0.12	-0.12
PC_42_3_	-0.02	-0.05	-0.09	-0.10	-0.11	0.07	0.25	0.14	0.02
PC_O_30_0_	-0.28	-0.05	0.06	-0.05	0.14	-0.13	-0.11	-0.05	-0.04
PC_O_32_1_	-0.10	-0.08	0.01	-0.09	0.06	-0.20	0.11	0.07	0.00
PC_O_36_2_	-0.12	-0.16	0.02	-0.08	-0.03	-0.19	0.08	-0.08	-0.08
PC_O_36_3_	0.01	-0.17	-0.05	-0.09	0.07	-0.20	0.39	0.23	0.12

PC_O_42_1_	-0.18	-0.02	0.18	0.30	0.08	0.03	-0.14	-0.28	-0.08
PC_O_42_2_	-0.25	0.00	0.23	0.26	0.09	0.11	-0.23	-0.33	-0.12
PE_34_2_	-0.17	-0.20	0.19	-0.14	-0.15	0.03	-0.14	-0.17	-0.07
PE_34_3_	-0.12	-0.16	0.15	-0.09	-0.14	0.04	-0.11	-0.08	-0.11
PE_36_2_	-0.15	-0.20	0.15	-0.18	-0.16	0.06	-0.09	-0.16	-0.04
PE_36_5_	-0.11	-0.14	0.05	-0.13	-0.21	-0.09	-0.11	-0.05	-0.23
PE_38_3_	-0.06	-0.21	0.04	-0.19	-0.19	-0.01	-0.02	-0.10	-0.12
PE_40_5_	-0.07	-0.21	0.00	-0.20	-0.15	-0.01	-0.07	-0.12	-0.15
Phenylalanine	0.01	-0.26	-0.11	-0.25	-0.08	-0.15	0.40	0.27	0.24
Phenylpyruvic_acid	-0.22	-0.15	-0.04	-0.11	0.20	-0.02	0.00	-0.02	0.14
PI_36_1_	-0.06	-0.22	-0.23	-0.20	-0.22	-0.16	0.07	0.05	-0.06
PI_36_3_	-0.05	-0.14	-0.27	-0.03	-0.19	-0.03	0.16	0.09	0.02
PI_40_7_	0.08	-0.14	-0.29	-0.24	-0.01	0.04	-0.08	-0.04	-0.20
PS_36_5_	0.07	-0.12	-0.22	-0.18	0.03	0.15	-0.16	-0.23	-0.21
PS_36_6_	-0.01	-0.08	-0.02	-0.13	-0.11	-0.15	0.03	0.03	-0.20
PS_40_5_	-0.04	-0.21	0.08	-0.03	0.00	0.20	-0.14	-0.26	-0.10
Pyroglutamic_acid	0.06	-0.24	-0.05	-0.24	-0.03	-0.07	0.33	0.34	0.23
Ribulose	-0.05	-0.36	0.25	-0.23	0.14	-0.09	0.23	0.19	0.07
Ricinoleic_acid	0.02	-0.20	0.18	-0.16	-0.02	-0.25	0.06	-0.11	0.00
SM_d16_1_16_0_	-0.16	-0.19	0.03	-0.18	0.05	-0.19	0.12	0.12	0.04
SM_d16_1_22_2_	-0.08	-0.32	0.10	-0.34	0.08	0.20	0.01	-0.19	0.18
SM_d18_0_14_0_	-0.19	-0.19	0.03	-0.17	0.06	-0.19	0.09	0.10	0.03
SM_d18_0_18_2_	-0.01	-0.37	0.08	-0.32	0.02	-0.13	0.13	-0.03	0.09
SM_d18_0_24_0_	-0.03	-0.21	-0.08	0.00	0.00	-0.21	0.23	0.13	0.02
SM_d18_0_26_0_	-0.09	-0.26	-0.01	-0.02	0.09	-0.21	0.17	0.14	0.05
SM_d18_0_26_2_	-0.14	-0.27	0.19	-0.12	0.04	-0.14	0.03	-0.01	0.04
Succinic_acid	0.06	-0.21	-0.02	-0.09	-0.14	-0.31	0.25	0.14	0.19
Tetradecanoylcarnitine	0.01	-0.23	0.06	-0.13	-0.11	-0.26	0.01	-0.21	-0.10
TG_45_0_	-0.17	-0.01	0.17	0.15	-0.05	0.05	-0.25	-0.20	0.03

TG_51_3_	0.07	-0.11	0.06	-0.06	-0.11	-0.12	0.25	0.30	0.22
TG_54_4_	0.00	-0.04	0.04	-0.20	0.00	-0.13	0.29	0.38	0.27
TG_54_5_	0.00	-0.04	0.04	-0.17	0.01	-0.10	0.24	0.31	0.26
TG_54_6_	0.02	-0.06	0.02	-0.15	0.06	-0.08	0.19	0.27	0.22
TG_54_7_	0.04	-0.04	0.02	-0.11	0.06	-0.08	0.23	0.31	0.21
TG_56_9_	0.05	-0.12	-0.10	-0.15	0.04	-0.06	0.27	0.32	0.07
Valine	-0.04	-0.26	-0.24	-0.16	0.12	-0.25	0.20	0.14	0.24
Xylose	-0.23	-0.18	0.27	-0.18	0.20	0.12	0.01	0.07	-0.08
Xylulose	0.19	0.11	-0.02	0.44	0.05	0.12	-0.11	-0.25	0.03

Note.—bUDCA, 3β-Ursodeoxycholic Acid; CA, cholic acid; CDCA, chenodeoxycholic acid; CER, Ceramide; DAG, Diacylglycero; EPA, Eicosapentaenoic acid; GCDCA_s, glycochenodeoxycholic Acid Sulfate; LPC, lysoPhosphatidylcholine; LPE, lysoPhosphatidylethanolamine; PE, Phosphatidylethanolamine; PI, Phosphatidylinositol; SM, Sphingomyelin; TG, Triacylglycerol.

Supplementary Table 9 Pearson correlation coefficients between CD-enriched bacteria genera and brain radiomics features

	Ctx-Lh-Superiortemporal vol	Ctx-Lh-Middle-Temporal vol	Left-Hippocampus R2Star p10	Ctx-Rh-Precentral fMRI ReHo mean	Brain-Stem fMRI ALFF p90	Ctx-Rh-Precentral fMRI ALFF p10	Ctx-Rh-Postcentral fMRI ReHo p90	Ctx-Lh-Cuneus fMRI ReHo std	Left-Putamen fMRI ReHo p10	Ctx-Lh-MiddleTemporal CBF p90	Left-Thalamus-Proper CBF std	Ctx-Rh-SuperiorFrontal DKI GA std	Ctx-Lh-Caudal-Anterior-Cingulate DKI KFA std
Actinomyces	0.04	0.10	-0.20	0.23	0.04	0.24	0.05	0.04	-0.19	0.08	-0.23	-0.18	0.39
Atopobium	0.09	0.13	-0.16	0.21	0.09	0.24	0.01	0.08	-0.20	0.26	0.03	-0.18	0.11
Collinsella	0.13	-0.22	0.11	-0.04	-0.02	0.21	0.06	0.35	-0.05	-0.14	-0.01	-0.02	0.04
Eggerthella	0.10	0.01	0.09	-0.01	-0.03	0.09	-0.09	0.01	-0.09	0.16	0.02	-0.03	0.00
Gemella	-0.05	0.17	-0.28	0.20	-0.02	0.16	0.09	0.01	-0.02	0.24	0.06	-0.15	-0.05
Granulicatella	-0.13	0.17	-0.25	0.24	0.10	0.27	0.01	-0.10	-0.19	0.29	-0.04	-0.16	0.06
Enterococcus	-0.11	0.11	-0.12	0.03	0.15	-0.26	-0.02	-0.07	-0.21	0.20	0.22	-0.08	-0.25
Lactobacillus	-0.02	-0.04	0.13	-0.18	-0.03	-0.06	0.12	0.23	0.09	-0.02	0.19	0.21	-0.03
Lachnoclostridium	0.02	0.04	0.21	-0.19	-0.17	-0.10	-0.20	-0.18	0.07	0.00	0.08	0.02	0.13
LachnospiraceaeUCG010	0.12	-0.15	0.06	-0.10	0.11	-0.06	-0.08	-0.04	0.06	-0.43	-0.30	-0.15	0.03
Oribacterium	-0.22	0.06	-0.16	0.14	0.13	0.22	-0.01	-0.05	-0.24	0.32	0.07	-0.15	-0.03
Tyzzerella4	0.15	0.08	0.06	-0.13	0.07	-0.21	-0.13	0.03	0.01	0.07	-0.05	-0.08	-0.01
Ruminococcusgnavusgroup	0.25	-0.03	-0.02	-0.13	-0.04	-0.01	-0.01	-0.08	-0.01	-0.18	-0.12	0.08	0.08
Ruminococcustorquesgroup	0.24	-0.11	-0.03	-0.07	-0.07	0.01	-0.16	-0.01	-0.09	-0.19	-0.18	-0.02	-0.13
Peptostreptococcus	0.17	0.11	-0.16	-0.07	0.13	-0.13	-0.21	-0.11	0.11	0.00	0.02	0.07	-0.09
Erysipelatoclostridium	0.12	0.13	-0.01	-0.10	-0.02	-0.17	0.00	-0.27	-0.09	-0.07	0.03	-0.13	-0.14
Faecalitalea	0.27	0.04	0.07	-0.03	-0.18	0.17	0.02	0.07	-0.22	0.01	-0.10	0.03	0.04
Solobacterium	0.03	0.13	-0.10	0.18	-0.03	0.13	-0.03	-0.03	-0.07	0.19	0.05	-0.09	-0.13
Clostridiuminnocuumgroup	0.06	0.08	0.10	-0.18	-0.16	-0.15	-0.08	-0.18	0.11	0.04	-0.05	-0.11	0.16

Veillonella	-0.10	0.25	-0.05	0.15	0.00	-0.05	-0.18	-0.20	-0.11	0.41	0.13	-0.08	-0.17
Fusobacterium	-0.02	0.00	0.05	-0.07	-0.13	-0.21	-0.23	-0.03	-0.19	-0.03	-0.06	-0.03	-0.13
Saccharimonadaceae	0.05	-0.01	-0.06	0.19	-0.12	0.28	0.17	0.08	-0.10	0.25	0.15	-0.18	-0.11
Saccharimonadales	-0.12	-0.05	0.00	0.05	0.10	0.19	0.03	0.03	0.03	0.32	0.06	-0.03	-0.10
EscherichiaShigella	0.08	0.06	0.07	0.13	-0.26	0.02	0.05	-0.02	0.19	-0.12	-0.01	0.04	-0.06

Note.—ctx, cortex; vol, volume; p10, 10th percentile; fMRI, functional MRI; ReHo, Regional Homogeneity; ALFF, Amplitude of Low-Frequency Fluctuations; p90, 90th percentile; STD, Standard Deviation; p10, 10th percentile; CBF, Cerebral Blood Flow; DKI, Diffusion Kurtosis Imaging; GA, Geodesic Anisotropy; KFA, Kurtosis Fractional Anisotropy.

Supplementary Table 10 definition and index introduction of Alpha and Beta diversity

Item	Computing method	Meaning of the item
**Alpha diversity**		Refers to the measures of species richness, diversity, and evenness within a local, homogeneous habitat, and is also known as within-habitat diversity.
**The Chao1 estimator index**	Originally proposed by Chao, this method estimates the actual number of species in a community by calculating the number of ASVs/OTUs detected only once (“Singleton”) and twice (“Doubleton”).	The larger the Chao1 index, the higher the richness of the community.
**Shannon diversity index**	The richness and evenness of the community were considered comprehensively.	A higher Shannon index or Simpson index indicates greater community diversity. The Shannon index is more sensitive to richness and rare ASVs/OTUs, making it suitable for complex communities, while the Simpson index is more sensitive to evenness and dominant ASVs/OTUs, making it more appropriate for simpler communities.
**The Simpson index**	The Simpson index, a common measure of community diversity, evaluates diversity by calculating the probability that two randomly sampled individuals (sequences) from the community belong to different species (ASVs/OTUs). A higher Simpson index value indicates greater community diversity.	
**Pielou’s Evenness index**	The evenness of a community is emphasized by dividing the Shannon index (H') by the natural logarithm of the total number of species (total ASVs/OTUs), which removes the richness effect of the Shannon index.	A higher Pielou's evenness index indicates a more uniform community.

**Beta diversity**		Beta diversity refers to the variation in species composition, or the rate of species turnover between different communities along environmental gradients. It is also known as between-habitat diversity.
**Bray–Curtis distances**	This weighted calculation method evaluates community similarity by computing the ratio of the sum of absolute differences in species abundances between two samples to their total abundances. It accounts for both the presence or absence of species and differences in their abundances.	For differences in microbial communities caused by environmental gradients or experimental treatments, which are often primarily due to changes in microbial abundances, weighted distance methods can be considered. Simultaneously, if evolutionary distance between microbes is not a key factor (i.e., greater evolutionary distance does not necessarily imply greater functional differences), Bray-Curtis distance can be used.

Supplementary Figures

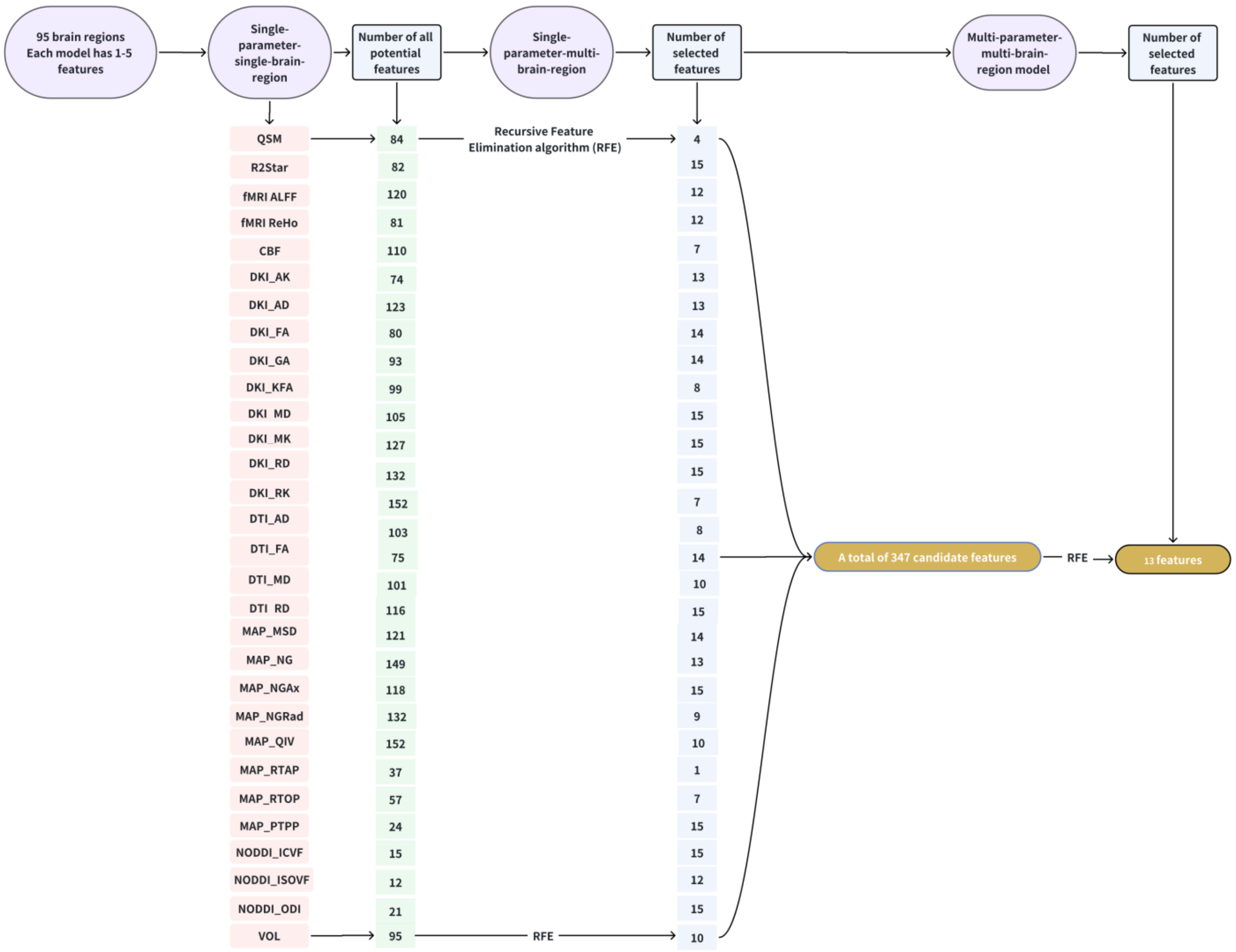

Supplementary Fig 1 Flow-chart of the feature selections steps.

(CBF, cerebral blood flow; QSM, quantitative susceptibility mapping; fMRI, functional MRI; ALFF, amplitude of low-frequency fluctuations; ReHo, regional homogeneity; DTI, diffusion tensor imaging; AD, axial diffusivity; FA, fractional anisotropy; MD, mean diffusivity; RD, radial diffusivity; NODDI, neurite orientation dispersion and density imaging; ICVF, intra-cellular volume fraction; ISOVF, isotropic volume fraction; ODI, orientation dispersion index; MAP, mean apparent propagator; MSD, mean squared displacement; NG, non-gaussian; NGAx, axial non-gaussian; NGRad, radial non-gaussian; QIV, q-space inverse variance; RTAP, return-to-axis probability; RTOP, return-to-origin probability; RTPP, return-to-plane probability; DKI, diffusion kurtosis imaging; AK, axial kurtosis; GA, geodesic anisotropy; KFA, kurtosis fractional anisotropy; MK, mean kurtosis; RK, radial kurtosis; RFE, recursive feature elimination.)

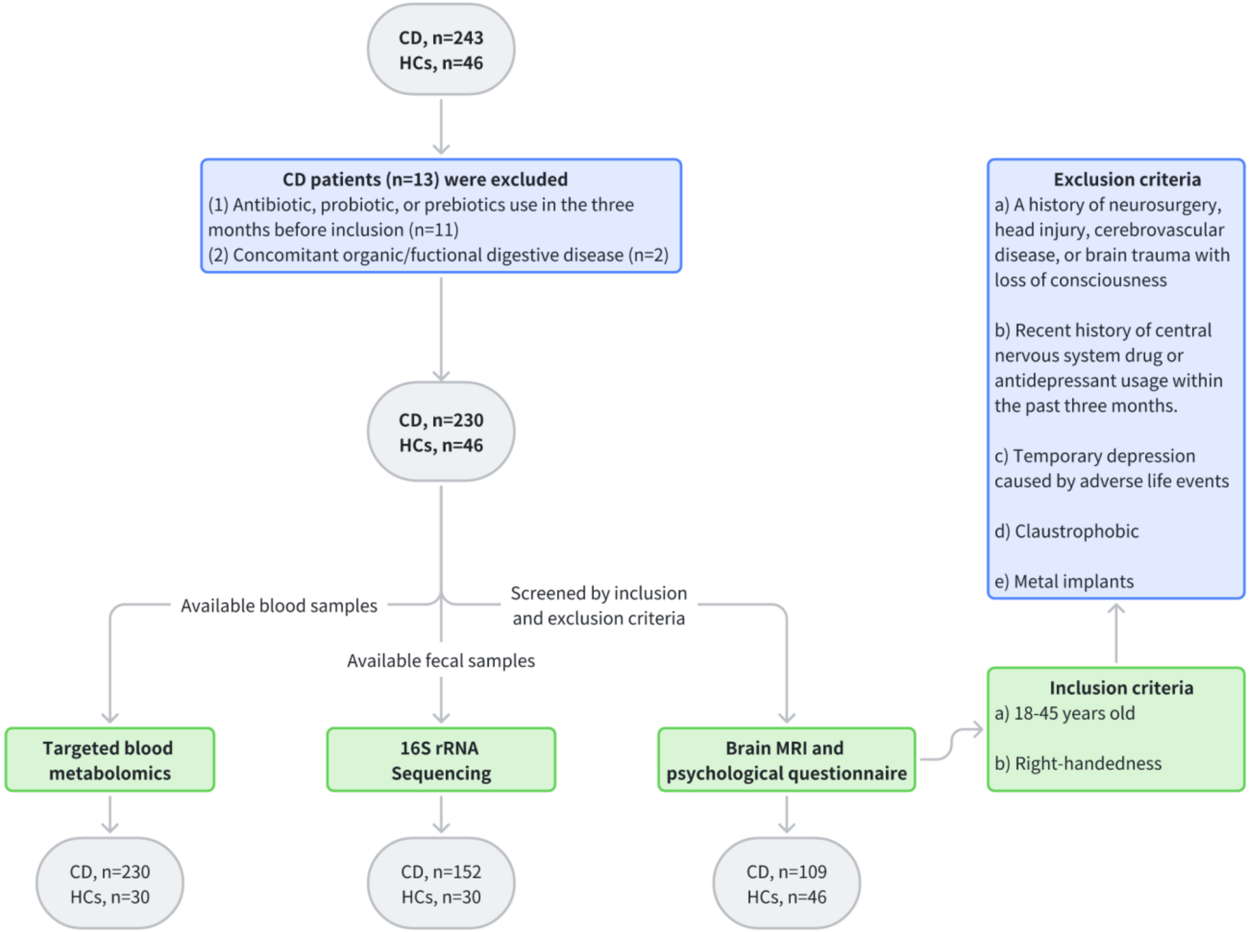

Supplementary Fig 2 Flowchart shows participants recruitment process.
(CD, Crohn’s disease; HCs, healthy controls; 16SrRNA, 16S ribosomal ribonucleic acid.)

Supplementary Fig 3 Chord diagram displaying the relationships among blood metabolites and CD-enriched genera

The chord diagram illustrates the relationships between 122 blood metabolites and nine CD-enriched genera in CD patients determined through linear regression analysis adjusting for age, sex, and body mass index (see **Supplementary Table 8**).

(AMP, adenosine monophosphate; CA, cholic acid; CDCA, chenodeoxycholic acid; CE, cholesteryl ester; CER, ceramide; DAG, diacylglycerol; DHA, docosahexaenoic acid; DPA, docosapentaenoic acid; EPA, eicosapentaenoic acid; GCDCA_s, glycochenodeoxycholic acid sulfate; LPC, lysophosphatidylcholine; LPE, lysophosphatidylethanolamine; PC, phosphatidylcholine; PE, phosphatidylethanolamine; PI, phosphatidylinositol; PS, phosphatidylserine; UDCA, ursodeoxycholic acid; SM, sphingomyelin; TG, triacylglycerol; bUDCA, 3β-ursodeoxycholic acid; DAG, diacylglycerol; SM, sphingomyelin.)

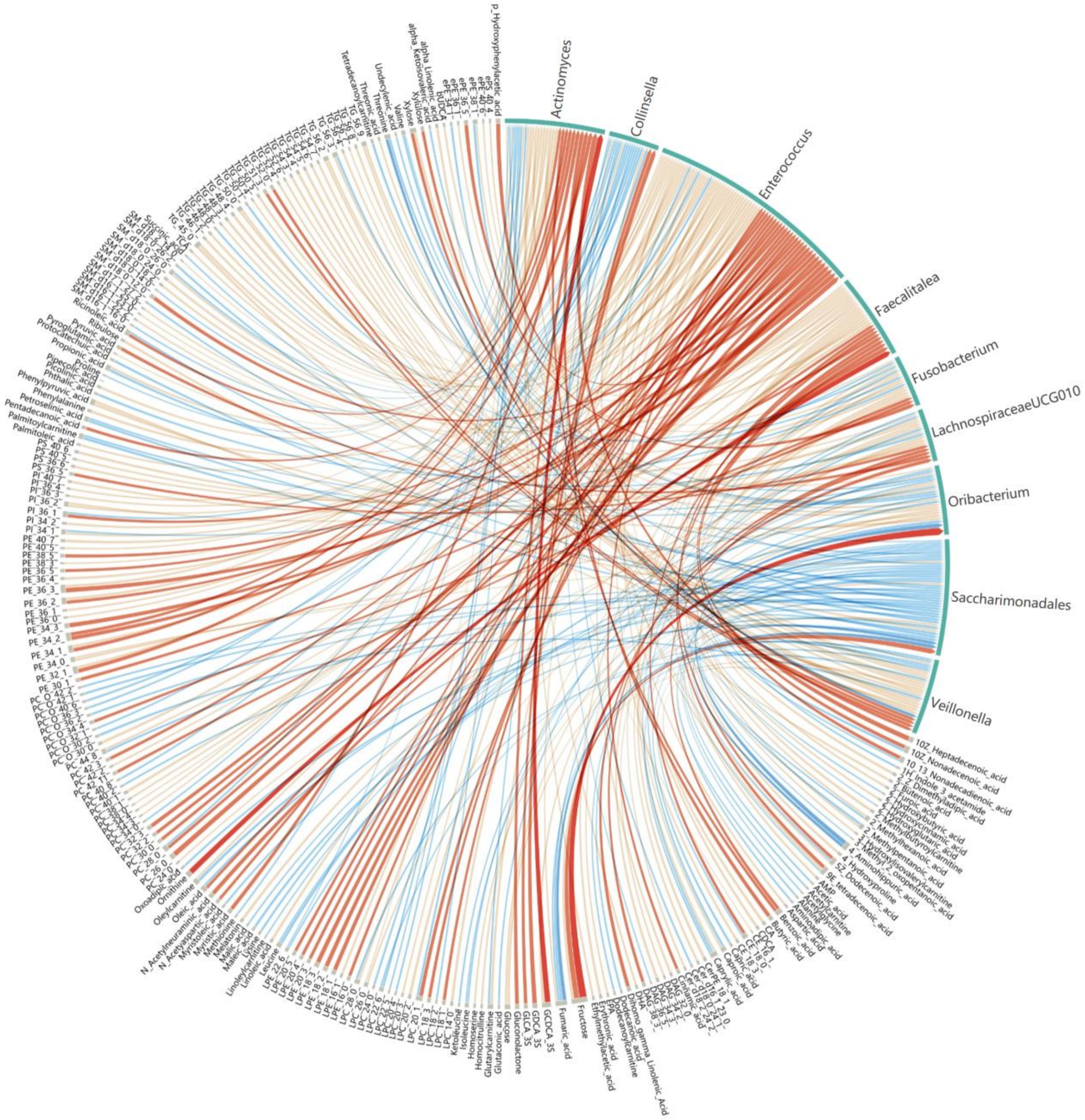

Supplement: Supplementary file 1 — ELECTRONIC SUPPLEMENTARY MATERIAL [file 13244_2024_1859_MOESM1_ESM.pdf]
